# Supplementary material for: Experiences of the clinical academic pathway: a qualitative study in Greater Manchester to improve the opportunities of minoritised clinical academics
Source: BMJ Open. 2024 Mar 19;14(3):e079759. doi: 10.1136/bmjopen-2023-079759 (PMC10973582; doi:10.1136/bmjopen-2023-079759)
Supplement: Supplementary data [file bmjopen-2023-079759supp001.pdf]

Interview questions, Version 1.1, 21/07/22

Supplement 1:

Indicative interview question stems – SCALE

Please note, the following are suggested, iterative question stems. The group interviews and individual interviews will both be semi-structured in nature, so interviewers will also be able to follow interesting lines of questioning/prompt further than the lists supplied below, if necessary. Interviewers will meet regularly to discuss interesting insights from the interviews, and will have the agency and ability to modify, evolve or add questions as appropriate, and as befits the iterative design of this qualitative research

Clinician academics focus groups

1. Tell us about your journey into clinical academia (Icebreaker question).
2. What facilitated your journey?
    a. (If appropriate): Why do you think you experienced such facilitators?
3. Did you experience any difficulties or barriers to your career?
    a. If so, please elaborate.
    b. Why do you think you experienced these barriers?
4. What is the research culture of your academic institution like?
5. Has the culture you've experienced at your academic institution influenced any decisions you've made about your career?
    a. If so, how?
6. Has the culture you've experienced within medicine/dentistry and academia more generally influenced any decisions you've made about your career?
    a. If so, how?
7. Thinking about any barriers or negative experiences of culture that have been mentioned, what changes could be made on an institutional level to enable clinical academics from minoritised backgrounds to flourish?
8. Is there anything else you wish to tell us about your experiences of clinical academia within Greater Manchester?

Clinicians focus groups

1. Tell us about your journey into clinical practice to date (Icebreaker question).
2. What facilitated your journey?
    a. (If appropriate): Why do you think you experienced such facilitators?
3. Did you experience any difficulties or barriers to your career?
    a. If so, please elaborate.
    b. Why do you think you experienced these barriers?
4. How do you view careers in clinical academia?
    a. Does anyone have experience as a clinical academic? If you left an academic post, can you tell us why?
    b. Have you ever considered a clinical academic career? If the answer is yes but you chose not to pursue a clinical academic career, can you tell us why?
    c. For those that haven't considered clinical academic careers, can you tell us why? Did anything in particular put you off?

Lin C-Y, *et al. BMJ Open* 2024; 14:e079759. doi: 10.1136/bmjopen-2023-079759

5. In Greater Manchester more specifically, do you get any sense of what it's like to be a clinical academic?
    a. What do you think about the culture surrounding research in Greater Manchester?
    b. Have your experiences or perceptions of this culture influenced the decisions you've made about your career in any ways?
6. Thinking about any barriers or negative experiences of culture that have been mentioned, what changes could be made on an institutional level to enable clinicians from minoritised backgrounds to pursue clinical academic careers, if they wish?
7. Is there anything else you wish to tell us about your experiences of the research culture or academia within Greater Manchester?

Medical and dental students focus groups

1. How familiar are you with the clinical academic career pathway? (Icebreaker question).
2. What kind of information, if any, have you received about clinical academic career during your studies?
3. Would you be interested in pursuing an academic career along with your clinical one?
4. Do you have an opinion about intercalated degrees?
5. Do you see difficulties or barriers to start a clinical academic career?
    a. If so, please elaborate.
    b. What do you think is the reason behind these barriers?
6. What is the institutional culture of the University of Manchester like?
7. Has the culture you've experienced at the university influenced any plans you've made about your career?
    a. If so, how?
8. Thinking about any barriers or negative experiences of culture that have been mentioned, what changes could be made on an institutional level to enable people from minoritised backgrounds to start a clinical academic career?
9. Is there anything else you wish to tell us about your experiences within the University of Manchester?

Gatekeeper interviews

1. Can you tell me about your current role? (Icebreaker question)
    a. Do you have/ What responsibilities do you have in terms of managing clinicians/clinical academics?
    b. Do you have/ What responsibilities do you have in terms of making selection decisions in clinical academia?
2. What have your experiences of managing clinicians/clinical academics and/or making selection decisions been like?
3. From your perspective, what are the facilitating factors for clinicians looking to build clinical academic careers?
4. From your perspective, what are the barriers for clinicians looking to build clinical academic careers?

5. Do you feel able to support the staff you manage to confront or tackle the barriers they may experience?
    a. If yes, can you give some examples of what this support looks like in practice? What resources does this support require?
    b. If no, can you please explain why?
6. If you have experience making selection decisions, do you feel able to confront any barriers to clinical academic careers in your role within selection?
    a. If yes, how?
    b. If no, why not? Could anything change to enable you to challenge the barriers you are aware of?
7. Thinking specifically about the experiences of those you manage who are from minoritised backgrounds, are there any additional facilitating factors for minoritised clinicians looking to build clinical academic careers?
8. Thinking specifically about the experiences of those you manage who are from minoritised backgrounds, are there any additional barriers for minoritised clinicians looking to build clinical academic careers?
9. Do you feel able to support the staff you manage from minoritised backgrounds to confront or tackle the barriers they may experience?
    a. If yes, can you give some examples of what this support looks like in practice? What resources does this support require?
    b. If no, can you please explain why?
