# Supplementary material for: Experiences of the clinical academic pathway: a qualitative study in Greater Manchester to improve the opportunities of minoritised clinical academics
Source: BMJ Open. 2024 Mar 19;14(3):e079759. doi: 10.1136/bmjopen-2023-079759 (PMC10973582; doi:10.1136/bmjopen-2023-079759)
Supplement: Author's manuscript [file bmjopen-2023-079759.draft_revisions.pdf]

BMJ Open

BMJ Open is committed to open peer review. As part of this commitment we make the peer review history of every article we publish publicly available.

When an article is published we post the peer reviewers' comments and the authors' responses online. We also post the versions of the paper that were used during peer review. These are the versions that the peer review comments apply to.

The versions of the paper that follow are the versions that were submitted during the peer review process. They are not the versions of record or the final published versions. They should not be cited or distributed as the published version of this manuscript.

BMJ Open is an open access journal and the full, final, typeset and author-corrected version of record of the manuscript is available on our site with no access controls, subscription charges or pay-per-view fees (http://bmjopen.bmj.com).

If you have any questions on BMJ Open's open peer review process please email info.bmjopen@bmj.com

BMJ Open

Experiences of the clinical academic pathway: A Greater Manchester study to improve the opportunities of minoritised clinical academics

Journal:	*BMJ Open*
Manuscript ID	bmjopen-2023-079759
Article Type:	Original research
Date Submitted by the Author:	11-Sep-2023
Complete List of Authors:	Lin, Chiu-Yi; The University of Manchester, Division of Pharmacy and Optometry, School of Health Sciences, Faculty of Biology, Medicine and Health<br>Greco, Cinzia; The University of Manchester, Centre for the History of Science, Technology and Medicine; The University of Manchester, Division of Medical Education, School of Medical Sciences, Faculty of Biology, Medicine and Health<br>Radhakrishnan, Hema; The University of Manchester, Division of Pharmacy and Optometry, School of Health Sciences, Faculty of Biology, Medicine and Health<br>Finn, Gabrielle; The University of Manchester, Division of Medical Education, School of Medical Sciences, Faculty of Biology, Medicine and Health<br>Cowen, Rachel; The University of Manchester, Division of Medical Education, School of Medical Sciences, Faculty of Biology, Medicine and Health<br>Gardiner, Natalie; The University of Manchester, Division of Diabetes, Endocrinology & Gastroenterology, School of Medical Sciences, Faculty of Biology, Medicine and Health
Keywords:	MEDICAL EDUCATION & TRAINING, QUALITATIVE RESEARCH, Sexual and Gender Minorities

SCHOLARONE™
Manuscripts

I, the Submitting Author has the right to grant and does grant on behalf of all authors of the Work (as defined in the below author licence), an exclusive licence and/or a non-exclusive licence for contributions from authors who are: i) UK Crown employees; ii) where BMJ has agreed a CC-BY licence shall apply, and/or iii) in accordance with the terms applicable for US Federal Government officers or employees acting as part of their official duties; on a worldwide, perpetual, irrevocable, royalty-free basis to BMJ Publishing Group Ltd ("BMJ") its licensees and where the relevant Journal is co-owned by BMJ to the co-owners of the Journal, to publish the Work in this journal and any other BMJ products and to exploit all rights, as set out in our licence.

The Submitting Author accepts and understands that any supply made under these terms is made by BMJ to the Submitting Author unless you are acting as an employee on behalf of your employer or a postgraduate student of an affiliated institution which is paying any applicable article publishing charge ("APC") for Open Access articles. Where the Submitting Author wishes to make the Work available on an Open Access basis (and intends to pay the relevant APC), the terms of reuse of such Open Access shall be governed by a Creative Commons licence – details of these licences and which Creative Commons licence will apply to this Work are set out in our licence referred to above.

Other than as permitted in any relevant BMJ Author's Self Archiving Policies, I confirm this Work has not been accepted for publication elsewhere, is not being considered for publication elsewhere and does not duplicate material already published. I confirm all authors consent to publication of this Work and authorise the granting of this licence.

For peer review only

Experiences of the clinical academic pathway: A Greater Manchester study to improve the opportunities of minoritised clinical academics.

ABSTRACT

Objectives: The aim of this study was to explore the barriers and facilitators faced by clinical academics in the Greater Manchester region, with particular attention to the experiences of minoritised groups.

Design: A qualitative study using semi-structured interviews and focus groups was conducted. A reflexive thematic analysis was applied to identify key themes.

Setting: University of Manchester and NHS Trusts in the Greater Manchester region.

Participants: The sample of this study was composed of 43 participants, including clinical academics, stakeholders, clinicians, and medical and dental students.

Results: Six themes were identified. Clinical academics face several barriers and facilitators, some of which – 1) funding insecurity and 2) high workload between the clinic and academia – are common to all the clinical academics. Other barriers, including 3) discrimination that translates into struggles with self-worth and feeling of not belonging, 4) being or being perceived as foreign; and 5) unequal distribution of care duties, particularly affect people from minoritised groups. In contrast, 6) mentorship was commonly identified as one of the most important facilitators.

Conclusions: Cultural and structural interventions are needed, such as introducing financial support for early career clinical academics and intercalating health care students to promote wider social and cultural change and increase the feelings of belonging and representation across the entire clinical academic pipeline.

For peer review only - http://bmjopen.bmj.com/site/about/guidelines.xhtml

STRENGTHS AND LIMITATIONS OF THIS STUDY

1. In-depth interviews with a broad range of participants were conducted to explore the multidimensional barriers faced by clinical academics and the role played by stakeholders in supporting them.
2. An innovative qualitative analysis combining thematic analysis and Extended Case Method was used to analyse and interpret the data.
3. Only doctors and dentists were interviewed, professionals from other allied health disciplines were excluded.
4. The data focus only on the experiences of professionals working in Greater Manchester.

Word count: 4621

For peer review only

INTRODUCTION

The diversity profile of clinicians who choose the clinical academic (CA) pathway is not representative of the demographics of either clinical professionals with similar entry qualifications or that of the general population that they serve. Furthermore, the CA data shows that there are systematic inequalities within CA careers as the level of seniority increases [1]. It is evident that the proportion of CA from minoritised backgrounds is still relatively low in the field. A survey in the UK found that only 33% of CA are female, ranging from only 13% to 58% in different specialities[2]. This also largely reflects women often having lower academic rank and few attaining a leadership role in clinical academia[3 4]. In terms of ethnic profiles, minoritised ethnic CA are even less represented: only 17% of CA identify as having Black, Asian or Minority Ethnic backgrounds[2].

The diversity in the medical academic workforce is pivotal to increase innovation in healthcare, deliver high quality patient care, and provide visible role models and mentoring support for students and early career professionals, especially for those from minoritised ethnic backgrounds[5 6]. This also brings benefits to public health such as reducing health inequalities and building long term trustful relationship with disadvantaged communities[7 8].

Various challenges have already been recognised for CA, such as limited funding, financial insecurity, limited CA positions, and the demanding workload of a clinical role[9-13]. For CA from minoritised backgrounds, those challenges could be compounded. For example, for female CA, social expectations around family commitment and parenting responsibilities could result in their being unable to pursue CA careers[9 14]. In some cases, parental leave is limited by gender-based discrimination and sexism in the work culture[9]. Barriers such as the under-representation of ethnic minority colleagues in workplaces, a lack of cultural competency and trust from people at senior levels contribute to the challenges for CA from ethnic minority groups[13 15].

Previous work has highlighted the value of intercalation for healthcare students to the diversity of the CA pipeline[11 16]. Interrupting professional training to develop the research skills in our early career future healthcare professionals is critical to widen access and participation for underrepresented groups in clinical academia. The removal of Educational Achievement

For peer review only - http://bmjopen.bmj.com/site/about/guidelines.xhtml

scores from the allocation process for foundation posts is a cause for alarm, as it will have far-reaching consequences for student uptake of intercalation and amplify the inequities in the clinical academic pipeline[17]. Significant investment is required to prevent further inequity at early career stages, such as funding and scholarships to students from disadvantaged backgrounds.

Increasing the diversity of the CA workforce is emphasised in policies internationally[18]. In the UK, the NHS Race Equality Action Plan presented a mandatory workforce race equality standard[19 20]. According to Kline et al.[21], in Northern England (which includes Greater Manchester) Ethnic minority representation at board and very senior management level was significantly lower than in the overall NHS workforce and also in the local communities served.[21] Therefore, some regional actions have been taken to tackle the situation. The Manchester Foundation Trust introduced 'the Trust's Equality, Diversity and Inclusion Strategy 2019-2023' to ensure a representative and supported workforce[22]. There has been an improvement in diversity of CA (gender and ethnicity) both in UK academic institutions and in the NHS[1 23]. Whilst there has been an improvement in the representation of CA, more work still needs to be done to attract and importantly sustain a diverse population of CA.

It is critical to understand the negative and positive experience of doctors and dentists from minoritised backgrounds, the barriers they experience and areas of support and institutional good practice in removing any systemic barriers. This data will inform recommendation at a local level which will also be applicable to a wider UK context.

METHODS

Design

A qualitative study using semi-structured interviews and focus groups was conducted to explore the experiences of the CA doctors and dentists in Greater Manchester, with a particular focus on those identifying as being from minoritised backgrounds (including but not limited to gender, race/ethnicity, and/or sexual orientation).

Setting and samples

For peer review only - http://bmjopen.bmj.com/site/about/guidelines.xhtml

Theoretical and snowball sampling were employed to recruit CA (doctors and dentists, in research-focused, teaching-focused, and research and teaching posts) and medical and dental students (years 3-5) from the University of Manchester, clinicians in Greater Manchester, key senior stakeholders and gatekeepers to CA and clinical posts in Greater Manchester. In order to capture the diversity of experiences, participants were recruited from a wide range of health settings, at various stages of their careers, and with an emphasis to include minoritised profiles.

Data collection

Potential participants were identified through the members of established medical education networks, clinical academic training and academic networks of the research team, and the snowball sampling approach involving already interviewed participants. Posters and social media platforms (such as Twitter and Faculty newsletters at the University of Manchester) were used to advertise the study. Any potential participants who were interested in taking part were able to contact the authors directly. All potential participants were provided with an online information sheet and gave fully informed consent before and during participation in the research.

Both focus group and individual interviews were carried out virtually through Zoom [5.13, Zoom Communications] or Microsoft Teams [1.5, Microsoft], lasting between 17 and 61 minutes. The interview topics included experiences of CA career pathway or stake holder perspective, barriers/facilitators encountered, especially for people from minoritised groups, and experiences of local research cultures. Brief field notes were taken for all interviews and focus groups to facilitate data analysis and identify the initial coding. All interviews and focus groups were conducted by first two authors (CL & CG). Data collection was undertaken from September 2022 to February 2023.

To ensure data saturation in this study, the authors continued data collection until the point at which no new codes or themes were generated from additional data.

For peer review only - http://bmjopen.bmj.com/site/about/guidelines.xhtml

Data analysis

All interviews and focus groups were digitally recorded, transcribed and analysed following Braun and Clarke's reflexive approach to thematic analysis[24]. To consider existing qualitative research on the careers of CA and the barriers faced by minoritised groups, the interpretation of the results was integrated with Burawoy's principle of 'reconstruction', in which anomalies in relation to an established theoretical corpus are considered and integrated in the analysis through the addition of new themes[25].

RESULTS

The study aimed to explore the experiences, barriers, and facilitators that CA (doctors and dentists) encounter in pursuing a CA career. A total of 43 participants took part in either interview or focus group for this study. In this study, 21 men and 22 women with a wide range of roles participated (19 clinical academics, 5 clinicians, 13 stakeholders and 6 medical or dental students). The 6 students were all in their 20s, the other participants were aged between 30 and 70 (9 in 30-39; 7 in 40-49; 18 in 50-59 and 3 in 60+). Of the 43 participants, 32 were White, 9 were Asian, and 2 were Black. The semi-structured interview framework focused on both the barriers and facilitators for minoritised groups and also general barriers and facilitators applicable to all CA.

Six main themes were generated: Theme 1: A highly competitive pathway that gives limited job security; Theme 2: Balancing two high-workload careers; Theme 3: Lack of representation and role models as limits to belonging; Theme 4: Being foreign or perceived as such; Theme 5: Childcare and gender inequalities; Theme 6: Support and recognition through mentorship. Of these themes, the first three describe barriers common to all CA, themes 3-5 describe barriers that impact in particular participants from minoritised groups, while theme 6 describes one of the main facilitating factors for all CA.

Theme 1: A highly competitive pathway that gives limited job security

Participants expressed a strong feeling of insecurity in relation to their own career as CA, describing CA as a more precarious pathway when compared to a clinical career. Participants at all levels of seniority recognised that there is fear while holding fixed-term positions of

For peer review only - http://bmjopen.bmj.com/site/about/guidelines.xhtml

losing one's jobs, and of being unable to control how long CA employment would be based on the success rate of obtaining grants and the duration of the grants.

Several participants mentioned the financial challenges of the CA pathway, including precarity and uncertainty linked to fixed-term contracts and limited funding. It was seen as problematic that CA needed to secure funding to continue in their career paths, particularly in the early stages of their career. One gatekeeper said:

> *"There is a lot of career uncertainty in academic life. I think it's becoming increasingly so since, say, in the last decade or so, because of funding difficulties in higher education. Determined junior members of staff may start out enthusiastically trying to cut a path in academic life, but I think they become frustrated at the lack of security in their job and it's all too easy to feel that you could move into a less rigorous discipline."* [G04]

Feelings that they were at risk of losing their jobs after the completion of the Academic Clinical Lecturer (ACL) training were widely reported by the participants. One said:

> *"I suppose the key step is if you're at the ACL level either with a formal ACL or trying to get your first independent fellowship, if that doesn't get funded by the time your ACL ends then just to earn a living you have to go back to the NHS, and then I think it's quite difficult. You've got to be very driven to be able to drive forward a fellowship application whilst you're still working full time in the clinical arena."* [G07]

One of the privileged routes to enter the CA pathway is through fellowships, and funding opportunities for fellowships are extremely competitive. One participant mentioned:

> *"There's a sort of a wish that everybody will go and get a very prestigious MRC [Medical Research Council] or Wellcome Trust fellowship, but actually there's not enough of those to go around."* [G06]

The participants who obtained the most prestigious fellowships and had straightforward careers (e.g. transiting from specialisation to academic clinical fellow and then lecturer progressing on

For peer review only

to a permanent CA position) also reported being concerned or anxious about finding their next job or securing their next grant in academia. The participants felt that continuous and secure employment was increasingly difficult to find. Therefore, uncertainty of subsequent employment and funding was also described as a major barrier to staying in the field. For example:

> *"As opposed to research, where you literally, if you don't have the next thing* [research job]*, it's like where is your next pay cheque coming from. So, also making that not a thing for people who are specifically research. I don't know how you would do that. It's just, yeah. I know there's no easy answer to some of these things."* [CA07]

Whilst all participants were keen to pursue CA career pathways, the uncertainty of employment as a CA emerged as a constant source of anxiety. As a result, the participants felt that they needed to prioritise and maintain an income to meet their basic living costs. A number of participants believed that a purely clinical career was more attractive due to its stability and clearly defined clinical progression route. One participant also explained:

> *"Academia does not support a family life, whereas the NHS does so. And when I speak to my peers who are in academia, that's what they say: they are just not supported. And the way that academia works, is that you're going from contract to contract and grants, no stability at all. So, I'm glad I'm not an academic for that reason."* [G08]

The importance of securing and sustaining funding was noted as a key challenge by a number of the participants. Supporting early and mid-career CA fellows through internal and external bridging funds whilst they are preparing for their next grant application was identified as a facilitator for career progression. One mid-career clinical academic shared the following experience:

> "*These things* [funding] *are very competitive. I was very fortunate: I got the *** Clinical Training Fellowship, the first I applied for, and I was successful, so that was fortunate. The Intermediate Fellowship took me a few goes and at the time there was no... there were no schemes to support clinical academics in that intermediate phase.*

For peer review only - http://bmjopen.bmj.com/site/about/guidelines.xhtml

And I think it's a bit better now because there's the NIHR clinical lectureship scheme that provides some reaching [bridging] *support, because you can't jump straight from a clinical train to a PhD and straight to an intermediate fellowship. So, I was very fortunate to be working in an environment where there was funding to support me, um, but that was a challenge."* [CA18]

Theme 2: Balancing two high-workload careers

Clinical academic careers can offer the benefits and rewards of making a difference to patients and having the space to pursue research projects. However, the challenge of how to balance an academic role with a clinical career was one of the common barriers mentioned:

"And I say to all clinicians who express an interest in academic work that everything is possible, but that you are embarking upon a career that has a clinical component and academic component, and you will be juggling, and it will affect the intensity of your work. So, people have the awareness that this isn't gonna be the easiest route." [G11]

The workload for both the clinical and academic roles often goes beyond what is anticipated based on the clinical academic job plan. Effective line management and responsive workload management between both roles can reduce stress and improve satisfaction whilst meeting the demands of both roles. From participants' reports, workloads go beyond the official hours both in the clinic and in academia. In addition, balancing research, teaching, and clinical roles was a common challenge:

"Two-and-a-half days were taken up by my clinical work, which is the same as was, and two days were taken up for teaching, so it's not quite accurate to say I had no research time, but I had half a day of research time in that five days and when you are a clinical academic you need to do lots of things to build up your name, especially when you're starting. You need time to go for grants. You need time to write papers. You need time to network and do increasing amounts and gain momentum." [CA09]

For peer review only - http://bmjopen.bmj.com/site/about/guidelines.xhtml

A common difficulty was the feeling of being considered as not doing enough work in the clinic compared to full-time colleagues, with the research work in some cases being considered less strenuous than the clinical work that CA are expected to do in the same measure as full-time colleagues. For junior clinicians this further could mean being potentially seen as someone not acquiring the necessary clinical skills, as in the case of this junior clinical academic:

> *"If we are not in theatre learning, we won't acquire our skills. Particularly from a clinical perspective it requires a lot of flexibility from trainers. These are surgeons, they just live, breathe, eat surgery. I am there 50% of the time. Sometimes they see me, sometimes they don't, and then they ask me 'are you here tomorrow?' You say 'no, I am not here'. You feel inadequate, you feel like you are not giving 100%."* [CA06]

Theme 3: Lack of representation and role models as limits to belonging

Barriers linked to systemic forms of discrimination can act on a more personal level, undermining a person's sense of self-worth and well-being. Sexism, racism, and other forms of discrimination that individuals experience throughout their life can impact their work life.

> *"But as an ethnic minority you feel those, you have grown up with them and some of them are not overt. Prejudices are, for me, I can usually sense them rather than someone* [...] *overtly saying no* [...] *You have heard it so many times, you kind of just think, right, okay, I am going to go and knock on a different door. I would say those things are the things that made me constantly more think I have to prove myself, I have to prove myself."* [CA06]

The importance of representation and a feeling of belonging is crucial for a diverse and dynamic workforce. However, it is common for some specialties, research fields, and/or senior leadership positions to be occupied by white British men. Many of the women participants have for example described leadership positions within academia as closed circles. Indeed, many of the women who did not think that their gender has negatively impacted on their career recognise that some higher roles are much more male-dominated.

For peer review only - http://bmjopen.bmj.com/site/about/guidelines.xhtml

"We still live in a world where they [men] *dominate senior academic and senior clinical and senior college and senior government type positions, and so they make a lot of decisions. I think that they occupy that space more than women, and that is levelling up all the time. I don't think they've ever stood in my way."* [CA11]

The lack of diverse role models and peers with whom it was possible to identify was discussed, along with the feeling of being out of place in different settings.

"If I go to a meeting of researchers […] *I might be the only South Asian in the room. That's when I feel like a minority; I never feel like a minority in clinical meetings."* [CA09]

"And even less so when you're black and woman, there's even less of that [ethnic minority women in CA] *And sometimes it's like, oh…can you do this?"* [CA07]

Greater Manchester is arguably one of the most diverse areas of the UK. The importance of diversifying the CA pipeline was recognised in the Trust's Equality, Diversity, and Inclusion Strategy 2019-2023 (introduced by Manchester University NHS Foundation Trust)[22] which led to actions such as improving diversity of interview panels. Some interviewees reported positive experiences, such as this female ethnic minority CA:

So, I went and did the interview and my interview panel actually was a group of all women, all different ethnicities and, I don't know, immediately I just felt way more at ease with them. I felt like I could be myself. We spent 45 minutes talking about me and my career goals and what I wanted and they really…I felt like they really got to know me and they gave me that time and respect to open up and answer questions. [CA04]

Theme 4: Being foreign or perceived as such

Along with the difficulties linked to race and ethnicity, some of the barriers discussed by the interviewees were more specifically linked to not being British-born or not being British citizens. Having a foreign accent was recognised as a marker of not being British-born, and

For peer review only

For peer review only - http://bmjopen.bmj.com/site/about/guidelines.xhtml

potentially non-British-trained. As a result, CA with the same ethnicity could have very different experiences. As one participant discusses:

> *"The other thing I think is something to do with how well you can speak and whether you have an unusual accent, which some people do, sometimes that accent can be taken as a bias against subconsciously, I feel. It hasn't happened to me, but I have seen other people who are very capable, but with a strong accent* […] *maybe could have done better."* [C04]

Language barriers, even if very modest, were also recognised as potentially creating a disadvantage in publishing and therefore building esteem and an academic career. Others underlined the role that discrimination can play, such as the peer review of grants and articles. Interviewees have underlined how in these cases the doubt remains whether a non-English name might negatively influence the outcome.

Theme 5: Childcare and gender inequalities

The most discussed barriers experienced along gender lines were linked to maternity and childcare and return to work following a period of maternity leave was described as difficult by a number of interviewees, particularly where the expectation was that work can resume without any disruptions.

> *"The one thing* [a supervisor] *said to me was 'you just need to make sure that your childcare is bomb-proof'. So, I don't know how you can manage to have childcare that is completely perfect"* [G11]

Although maternity leave is recognised when accounting for career breaks in the academic CV, a number of interviewees felt that interruptions for maternity were not taken into account when considering the track record of candidates for promotion and career progression. Maternity was recognised as a source of discrimination even by women who did not have children.

Childcare was further linked by several participants to barriers for the CA careers. A number of interviewees discussed how childcare continues to largely fall upon women, and how it is

For peer review only - http://bmjopen.bmj.com/site/about/guidelines.xhtml

difficult to reconcile the already high workload of clinic, research and/or teaching with parenting. The difficulty of managing the different workloads combined with the demands in terms of flexibility and potential need to relocate linked to the academic career. As a result, a number of interviewees observed how a purely clinical career was more attractive as it was more stable with less workload outside hospital hours, and how this might be an incentive to abandon the CA pathway.

Theme 6: Support and recognition through mentorship

Mentorship was identified as a main facilitator for CA career development. Mentorship relationships were mostly built through personal contacts, professional networks, or formal mentorship programs. Typically, the participants were more likely to seek mentorship from people who have the same or similar life experiences and were successful in CA. The participants believed that supportive mentorship could facilitate exploring their research interests, building their portfolio and expanding their professional networks. Mentors were able to provide deep insight into CA career pathways, for example:

> *"The person who's helped me the most was my PhD supervisor. He is a clinical academic; he interviewed me the first time round and didn't give me the fellowship, but encouraged me to seek other opportunities. He knew… because he'd been through it himself, he was aware of the trajectory, he was aware of what was needed, he really helped me in terms of how to write a fellowship application, how to sell yourself, really, which I think is something that I struggle with. So he's been very instrumental in helping my career, definitely, and seeing that I potentially have a future in this."* [CA03]

Participants who self-identified as earlier career researchers, pointed out the importance of senior and peer mentors in CA career pathways. Beyond the academic support, some participants also noted that mentors are pivotal when they encounter challenges in the field. The benefits of mental or emotional support from these mentors were reported. Based on their advanced knowledge or experience, mentors were in a good position to offer advice on CA career pathways. The participants felt they were protected: in a safe place. They believed these mentors could help them to develop into professional and independent CA, for example:

For peer review only

For peer review only - http://bmjopen.bmj.com/site/about/guidelines.xhtml

"I had a fantastic mentor […] *and she kind of took me under her wing. She could see I felt a bit lost. I didn't get the training number. I was, you know, five hours away from my family and she kind of just gave me this motivation. And some focus to say, 'Right. You're next. I know it's difficult for you up here and you're lonely, but you need to focus on the next step.' And she was pivotal really, in that she really believed in me. Like, really, for somebody, I didn't know very well. I don't know. She had this incredible ability to make me quite confident in my skills."* [CA06]

DISCUSSION

The participants to our research project form a diverse group including CA, clinicians, students and stakeholders, which has made it possible to understand a wide range of difficulties and problems impacting decision-making and career choices. Six themes have been identified, with two describing general barriers experienced by CA, three capturing further the complex experiences of minoritised groups, and one identifying an important facilitator.

This project has corroborated previous research [9 10] in showing the difficulties in managing two demanding careers and in finding a balance for activities with a significant workload that are difficult to scale to part-time positions, and how this creates challenges for career advancement, especially for people with childcare responsibilities.

Several new elements emerge from the analysis of our data. Among the general barriers, our participants mentioned recent changes in the career pathway, including the reduction of available positions, coupled with the expectation of obtaining prestigious fellowships before advancing in CA careers. The participants highlighted the degree to which obtaining such fellowships requires time, support from experienced colleagues, and availability of bridge funding to allow time to reapply in case of rejection. While available in the context studied, such resources are unequally distributed, and require in turn a significant time and financial investment for an uncertain outcome. Further, representation and inclusion need to improve, to bolster the feeling of belonging for minoritised CA.

One of the main individual barriers are the ways in which being minoritised hinders one's confidence. Ethnic minority participants reported episodes of racism, in their careers, but

For peer review only - http://bmjopen.bmj.com/site/about/guidelines.xhtml

further discussed how experiences of discrimination and racism suffered in other sphere of the life can have an impact also on their self-worth in professional settings. The feeling of unbelonging further erodes confidence. For example, women participants perceived men occupying the majority of the senior and management positions as disempowering. Despite improvement in female representation in leadership positions, gender imbalance can impact career progression through the phenomena already identified as glass ceiling and/or sticky floors [9 10]. Ethnic minority participants have reported similar experiences, with further issues such as having a name perceived as foreign and being potentially disadvantaged in being evaluated for publications and grants.

Taken together these data highlight the range of systemic barriers experienced by minoritised CA. These ranged from how having an accent was perceived as limiting progression, to lack of role models, difficulties in balancing childcare commitments and two highly demanding roles. The study also highlighted key enablers through bridging financial support, grant writing support and recognising lived experience and supporting career progression through mentorship. These findings will help inform individuals, senior leaders and organisations of the facilitators and barriers that impact on CA career progression and help policy development for those leading on clinical academic training programmes and delivery of a targeted equality and diversity strategy.

There are limitations to this study. Although the purpose of qualitative studies is not to obtain statistical generalisability, the relatively small number of respondents and the fact that only a quarter of them were from minoritised groups should be taken into account in the analysis of the results. Further studies with wider and more diverse samples might be helpful to enhance transferability in qualitative research. Meanwhile, as one aim of this study was to understand the experiences of minoritised groups, the composition of the research team should also be considered. Our team is diverse in terms of ethnicity and national origin, but is entirely female, which can have had a role in the collection and analysis of the data.

CONCLUSIONS

The study showed that there are several barriers that current and prospective CA face. It has also shown that barriers and difficulties are increased for participants from minoritised groups.

For peer review only

For peer review only - http://bmjopen.bmj.com/site/about/guidelines.xhtml

To remove these barriers, cultural and structural interventions are needed. On the one hand, it is important to identify forms of financial support for early career CA, in order to make the CA pathway more financially stable. On the other hand, it is important to encourage wider social and cultural changes that can reduce the feelings of unbelonging that women and ethnic minority participants in particular have expressed. These findings and recommendations are of relevance for senior leaders and organisations involved in clinical academic training and more general in equality & diversity and careers strategy.

For peer review only

For peer review only - http://bmjopen.bmj.com/site/about/guidelines.xhtml

Acknowledgments

Thanks to Prof Colette Fagan and Prof Adam Danquah, University of Manchester for helpful discussions during the course of this project. Thanks to clinicians, clinical academics, students and stakeholders who participated or facilitated this study.

Contributors

HR and NG conceived of and led the study as principal investigators; CL and CG conducted data collection and data analysis; GF, HR and NG contributed qualitative data analysis. All authors made contributions to the manuscript and reviewed the final submission.

Funding: This work was supported by the Wellcome Trust, grant number 204796/Z/16/Z ISSF 3-EDI and University of Manchester match funding

Declaration of Interest: None

Patient and public involvement

Patients and/or the public were not involved in the design, or conduct, or reporting, or dissemination plans of this research.

Patient consent for publication

Not applicable.

Ethics approval

This study involves human participants and was approved by the University of Manchester Ethics Committee (Reference: 2022-13791-23819). Informed consent was obtained from all participants in the study before taking part.

Data availability statement

The interview and focus group data used in this study are not publicly available due to integrity statements in the ethics approvals.

For peer review only

For peer review only - http://bmjopen.bmj.com/site/about/guidelines.xhtml

REFERENCES

1. Medical Schools Council. Clinical academic survey [Available from: https://www.medschools.ac.uk/clinical-academic-survey accessed 10/04 2023.
2. Nicole W. Survey of Medical Clinical Academic Staffing Levels London: Medical Schools Council; 2018 [Available from: https://www.medschools.ac.uk/media/2491/msc-clinical-academic-survey-report-2018.pdf2023.
3. Linden JA, Baird J, Madsen TE, et al. Diversity of leadership in academic emergency medicine: Are we making progress? *The American Journal of Emergency Medicine* 2022;57:6-13. doi: https://doi.org/10.1016/j.ajem.2022.04.009
4. Richter KP, Clark L, Wick JA, et al. Women physicians and promotion in academic medicine. *New England Journal of Medicine* 2020;383(22):2148-57. doi: 10.1056/NEJMsa1916935
5. Parker RB, Stack SJ, Schneider SM. Why diversity and inclusion are critical to the American college of emergency physicians' future success. *Annals of Emergency Medicine* 2017;69(6):714-17. doi: 10.1016/j.annemergmed.2016.11.030 [published Online First: 2017/02/01]
6. Alsan M, Garrick O, Graziani G. Does diversity matter for health? Experimental evidence from Oakland. *American Economic Review* 2019;109(12):4071-111. doi: 10.1257/aer.20181446
7. Beebeejaun K, Littleford K. A diverse public health workforce is more important than ever. *BMJ (Clinical research ed)* 2023;380:p447. doi: 10.1136/bmj.p447
8. Marmot M. Building back fairer in Greater Manchester and the country. Royal Society Open Science 2021;8(10):211454. doi: doi:10.1098/rsos.211454
9. Ranieri VF, Barratt H, Rees G, et al. A qualitative study of the influences on clinical academic physicians' postdoctoral career decision making. *Academic Medicine* 2018;93(11):1686-93. doi: 10.1097/acm.0000000000002141 [published Online First: 2018/01/26]
10. Trusson D, Rowley E, Bramley L. A mixed-methods study of challenges and benefits of clinical academic careers for nurses, midwives and allied health professionals. *BMJ Open* 2019;9(10):e030595. doi: 10.1136/bmjopen-2019-030595
11. Raine G, Evans C, Uphoff EP, et al. Strengthening the clinical academic pathway: a systematic review of interventions to support clinical academic careers for doctors and dentists. *BMJ Open* 2022;12(9):e060281. doi: 10.1136/bmjopen-2021-060281
12. Kehoe A, Crampton P, Buchanan J, et al. Tips to Support the Recruitment, Retention, and Progression of Clinical Academics. *Medical Science Educator* 2022;32(2):503-09. doi: 10.1007/s40670-022-01512-1
13. Finn G, Morgan J. From the sticky floor to the glass ceiling and everything in between: A systematic review and qualitative study focusing on gender inequalities in Clinical Academic careers, 2020.
14. Vassie C, Smith S, Leedham-Green K. Factors impacting on retention, success and equitable participation in clinical academic careers: a scoping review and meta-thematic synthesis. *BMJ Open* 2020;10(3):e033480. doi: 10.1136/bmjopen-2019-033480
15. Woolf K, Rich A, Viney R, et al. Perceived causes of differential attainment in UK postgraduate medical training: a national qualitative study. *BMJ Open* 2016;6(11):e013429. doi: 10.1136/bmjopen-2016-013429
16. Finn GM, Crampton P, Buchanan JA, et al. The impact of the COVID-19 pandemic on the research activity and working experience of clinical academics, with a focus on gender

For peer review only - http://bmjopen.bmj.com/site/about/guidelines.xhtml

and ethnicity: a qualitative study in the UK. *BMJ Open* 2022;12(6):e057655. doi: 10.1136/bmjopen-2021-057655
17. Finn GM, Crampton PES, Kehoe A, et al. Will removing intercalation undermine our clinical academic foundations? *BMJ (Clinical research ed)* 2021;374:n1527. doi: 10.1136/bmj.n1527
18. Boatright DH, Samuels EA, Cramer L, et al. Association between the Liaison Committee on Medical Education's Ddversity standards and changes in percentage of medical student sex, race, and ethnicity. *Jama* 2018;320(21):2267-69. doi: 10.1001/jama.2018.13705 [published Online First: 2018/12/05]
19. Priest N, Esmail A, Kline R, et al. Promoting equality for ethnic minority NHS staff—what works? *BMJ : British Medical Journal* 2015;351:h3297. doi: 10.1136/bmj.h3297
20. Ovseiko PV, Taylor M, Gilligan RE, et al. Effect of Athena SWAN funding incentives on women's research leadership. *BMJ (Clinical research ed)* 2020;371:m3975. doi: 10.1136/bmj.m3975
21. Kline R, Naqvi H, Razaq SA, et al. NHS Workforce Race Equality Standard: 2016 Data Analysis Report for NHS Trusts 2017 [Available from: https://www.england.nhs.uk/wp-content/uploads/2017/03/workforce-race-equality-standard-data-report-2016.pdf accessed 20 May 2023.
22. Equality, Diversity and Inclusion: Manchester Foundation Trust; 2023 [Available from: https://mft.nhs.uk/the-trust/equality-diversity-and-inclusion/ accessed 10/04 2023.
23. NHS Workforce Race Equality Standard 2022 [Available from: https://www.england.nhs.uk/wp-content/uploads/2022/04/Workforce-Race-Equality-Standard-report-2021-.pdf accessed 10/04 2023.
24. Braun V, Clarke V. Reflecting on reflexive thematic analysis. *Qualitative Research in Sport, Exercise and Health* 2019;11(4):589-97. doi: 10.1080/2159676X.2019.1628806
25. Vila-Henninger LA. Turning talk into "rationales": Using the extended case method for the coding and analysis of semi-structured Interview data in ATLAS.ti. *Bulletin of Sociological Methodology/Bulletin de Méthodologie Sociologique* 2019;143(1):28-52. doi: https://doi.org/10.1177/0759106319852887

For peer review only - http://bmjopen.bmj.com/site/about/guidelines.xhtml

Standards for Reporting Qualitative Research (SRQR)*

http://www.equator-network.org/reporting-guidelines/srqr/

Page/line no(s).

Title and abstract

**Title** - Concise description of the nature and topic of the study Identifying the study as qualitative or indicating the approach (e.g., ethnography, grounded theory) or data collection methods (e.g., interview, focus group) is recommended	Page 1
**Abstract** - Summary of key elements of the study using the abstract format of the intended publication; typically includes background, purpose, methods, results, and conclusions	Page 1

Introduction

**Problem formulation** - Description and significance of the problem/phenomenon studied; review of relevant theory and empirical work; problem statement	Page 3
**Purpose or research questio**n - Purpose of the study and specific objectives or questions	Page 4

Methods

**Qualitative approach and research paradigm** - Qualitative approach (e.g., ethnography, grounded theory, case study, phenomenology, narrative research) and guiding theory if appropriate; identifying the research paradigm (e.g., postpositivist, constructivist/ interpretivist) is also recommended; rationale**	Page 4-5
**Researcher characteristics and reflexivity** - Researchers' characteristics that may influence the research, including personal attributes, qualifications/experience, relationship with participants, assumptions, and/or presuppositions; potential or actual interaction between researchers' characteristics and the research questions, approach, methods, results, and/or transferability	Page 15
**Context** - Setting/site and salient contextual factors; rationale**	Page 4-5
**Sampling strategy** - How and why research participants, documents, or events were selected; criteria for deciding when no further sampling was necessary (e.g., sampling saturation); rationale**	Page 4-5
**Ethical issues pertaining to human subjects** - Documentation of approval by an appropriate ethics review board and participant consent, or explanation for lack thereof; other confidentiality and data security issues	Page 16
**Data collection methods** - Types of data collected; details of data collection procedures including (as appropriate) start and stop dates of data collection and analysis, iterative process, triangulation of sources/methods, and modification of procedures in response to evolving study findings; rationale**	Page5

For peer review only - http://bmjopen.bmj.com/site/about/guidelines.xhtml

For peer review only

**Data collection instruments and technologies** - Description of instruments (e.g., interview guides, questionnaires) and devices (e.g., audio recorders) used for data collection; if/how the instrument(s) changed over the course of the study	Page 5
**Units of study** - Number and relevant characteristics of participants, documents, or events included in the study; level of participation (could be reported in results)	Page 6
**Data processing** - Methods for processing data prior to and during analysis, including transcription, data entry, data management and security, verification of data integrity, data coding, and anonymization/de-identification of excerpts	Page 5
**Data analysis** - Process by which inferences, themes, etc., were identified and developed, including the researchers involved in data analysis; usually references a specific paradigm or approach; rationale**	Page 6
**Techniques to enhance trustworthiness** - Techniques to enhance trustworthiness and credibility of data analysis (e.g., member checking, audit trail, triangulation); rationale**	Page 5

Results/findings

**Synthesis and interpretation** - Main findings (e.g., interpretations, inferences, and themes); might include development of a theory or model, or integration with prior research or theory	Page 6
**Links to empirical data** - Evidence (e.g., quotes, field notes, text excerpts, photographs) to substantiate analytic findings	Page 6-14

Discussion

**Integration with prior work, implications, transferability, and contribution(s) to the field -** Short summary of main findings; explanation of how findings and conclusions connect to, support, elaborate on, or challenge conclusions of earlier scholarship; discussion of scope of application/generalizability; identification of unique contribution(s) to scholarship in a discipline or field	Pag 14-15
**Limitations** - Trustworthiness and limitations of findings	Page 15

Other

**Conflicts of interest** - Potential sources of influence or perceived influence on study conduct and conclusions; how these were managed	Page 17
**Funding** - Sources of funding and other support; role of funders in data collection, interpretation, and reporting	Page 17

*The authors created the SRQR by searching the literature to identify guidelines, reporting standards, and critical appraisal criteria for qualitative research; reviewing the reference lists of retrieved sources; and contacting experts to gain feedback. The SRQR aims to improve the transparency of all aspects of qualitative research by providing clear standards for reporting qualitative research.

For peer review only

For peer review only - http://bmjopen.bmj.com/site/about/guidelines.xhtml

**The rationale should briefly discuss the justification for choosing that theory, approach, method, or technique rather than other options available, the assumptions and limitations implicit in those choices, and how those choices influence study conclusions and transferability. As appropriate, the rationale for several items might be discussed together.

Reference:

O'Brien BC, Harris IB, Beckman TJ, Reed DA, Cook DA. **Standards for reporting qualitative research: a synthesis of recommendations.** *Academic Medicine*, Vol. 89, No. 9 / Sept 2014 DOI: 10.1097/ACM.0000000000000388

For peer review only

For peer review only - http://bmjopen.bmj.com/site/about/guidelines.xhtml

Experiences of the clinical academic pathway: A qualitative study in Greater Manchester to improve the opportunities of minoritised clinical academics.

Journal:	*BMJ Open*
Manuscript ID	bmjopen-2023-079759.R1
Article Type:	Original research
Date Submitted by the Author:	01-Dec-2023
Complete List of Authors:	Lin, Chiu-Yi; The University of Manchester, Division of Pharmacy and Optometry, School of Health Sciences, Faculty of Biology, Medicine and Health<br>Greco, Cinzia; The University of Manchester, Centre for the History of Science, Technology and Medicine; The University of Manchester, Division of Medical Education, School of Medical Sciences, Faculty of Biology, Medicine and Health<br>Radhakrishnan, Hema; The University of Manchester, Division of Pharmacy and Optometry, School of Health Sciences, Faculty of Biology, Medicine and Health<br>Finn, Gabrielle; The University of Manchester, Division of Medical Education, School of Medical Sciences, Faculty of Biology, Medicine and Health<br>Cowen, Rachel; The University of Manchester, Division of Medical Education, School of Medical Sciences, Faculty of Biology, Medicine and Health<br>Gardiner, Natalie; The University of Manchester, Division of Diabetes, Endocrinology & Gastroenterology, School of Medical Sciences, Faculty of Biology, Medicine and Health
**Primary Subject Heading**:	Medical education and training
Secondary Subject Heading:	Qualitative research
Keywords:	MEDICAL EDUCATION & TRAINING, QUALITATIVE RESEARCH, Sexual and Gender Minorities

I, the Submitting Author has the right to grant and does grant on behalf of all authors of the Work (as defined in the below author licence), an exclusive licence and/or a non-exclusive licence for contributions from authors who are: i) UK Crown employees; ii) where BMJ has agreed a CC-BY licence shall apply, and/or iii) in accordance with the terms applicable for US Federal Government officers or employees acting as part of their official duties; on a worldwide, perpetual, irrevocable, royalty-free basis to BMJ Publishing Group Ltd ("BMJ") its licensees and where the relevant Journal is co-owned by BMJ to the co-owners of the Journal, to publish the Work in this journal and any other BMJ products and to exploit all rights, as set out in our licence.

The Submitting Author accepts and understands that any supply made under these terms is made by BMJ to the Submitting Author unless you are acting as an employee on behalf of your employer or a postgraduate student of an affiliated institution which is paying any applicable article publishing charge ("APC") for Open Access articles. Where the Submitting Author wishes to make the Work available on an Open Access basis (and intends to pay the relevant APC), the terms of reuse of such Open Access shall be governed by a Creative Commons licence – details of these licences and which Creative Commons licence will apply to this Work are set out in our licence referred to above.

Other than as permitted in any relevant BMJ Author's Self Archiving Policies, I confirm this Work has not been accepted for publication elsewhere, is not being considered for publication elsewhere and does not duplicate material already published. I confirm all authors consent to publication of this Work and authorise the granting of this licence.

For peer review only

Experiences of the clinical academic pathway: A qualitative study in Greater Manchester to improve the opportunities of minoritised clinical academics.

Chiu-Yi Lin[1], Cinzia Greco[2,3], Hema Radhakrishnan[1], Gabrielle M Finn[3], Rachel L Cowen[3] and Natalie J Gardiner[4]

[1] *Division of Pharmacy and Optometry, School of Health Sciences, Faculty of Biology, Medicine and Health, University of Manchester, Manchester, M13 9PL, United Kingdom*

[2] *Centre for the History of Science, Technology and Medicine, University of Manchester, Manchester, M13 9PL, United Kingdom*

[3] *Division of Medical Education, School of Medical Sciences, Faculty of Biology, Medicine and Health, The University of Manchester, Manchester, M13 9PL, United Kingdom*

[4] *Division of Diabetes, Endocrinology & Gastroenterology, School of Medical Sciences, Faculty of Biology, Medicine and Health, University of Manchester, University of Manchester, Manchester, M13 9PL, United Kingdom*

Correspondence to Dr Hema Radhakrishnan
Carys Bannister Building,
University of Manchester,
Manchester M13 9PL
Email: Hema.Radhakrishnan@manchester.ac.uk
Tel: (+44)1613068763
Fax: (+44) 161 306 3969

Keyword: Medical education & training; Sexual and Gender Minorities; Qualitative Research

Word count: 5060

For peer review only

For peer review only - http://bmjopen.bmj.com/site/about/guidelines.xhtml

ABSTRACT

Objectives: The aim of this study was to explore the barriers and facilitators faced by clinical academics in the Greater Manchester region, with particular attention to the experiences of minoritised groups.

Design: A qualitative study using semi-structured interviews and focus groups was conducted. A reflexive thematic analysis was applied to identify key themes.

Setting: University of Manchester and NHS Trusts in the Greater Manchester region.

Participants: The sample of this study was composed of 43 participants, including clinical academics, stakeholders, clinicians, and medical and dental students.

Results: Six themes were identified. Clinical academics face several barriers and facilitators, some of which – 1) funding insecurity and 2) high workload between the clinic and academia – are common to all the clinical academics. Other barriers, including 3) discrimination that translates into struggles with self-worth and feeling of not belonging, 4) being or being perceived as foreign; and 5) unequal distribution of care duties, particularly affect people from minoritised groups. In contrast, 6) mentorship was commonly identified as one of the most important facilitators.

Conclusions: Cultural and structural interventions are needed, such as introducing financial support for early career clinical academics and intercalating health care students to promote wider social and cultural change and increase the feelings of belonging and representation across the entire clinical academic pipeline.

For peer review only

STRENGTHS AND LIMITATIONS OF THIS STUDY

1. In-depth interviews with a broad range of participants were conducted to explore the multidimensional barriers faced by clinical academics and the role played by stakeholders in supporting them.

2. An innovative qualitative analysis combining thematic analysis and Extended Case Method was used to analyse and interpret the data.

3. Only doctors and dentists were interviewed, professionals from other allied health disciplines were excluded.

4. The data focus only on the experiences of professionals working in Greater Manchester.

For peer review only

For peer review only - http://bmjopen.bmj.com/site/about/guidelines.xhtml

INTRODUCTION

The diversity profile of clinicians who choose the clinical academic (CA) pathway is not representative of the demographics of either clinical professionals with similar entry qualifications or that of the general population that they serve. Furthermore, the CA data shows that there are systematic inequalities within CA careers as the level of seniority increases (1). It is evident that the proportion of CA from minoritised backgrounds is still relatively low in the field. A survey in the UK found that only 33% of CA are female, ranging from only 13% to 58% in different specialities (2). This also largely reflects women often having lower academic rank and few attaining a leadership role in clinical academia (3, 4). In terms of ethnic profiles, minoritised ethnic CA are even less represented: only 17% of CA identify as having Black, Asian or Minority Ethnic backgrounds (2).

A clinical academic is a qualified healthcare professional who is engaged with clinical responsibilities in a healthcare setting whilst simultaneously holding an academic position and contributing to either research and teaching, or both. There is a well-defined career pathway for clinical academics with intercalation, based on clinical experience and academic qualification (https://www.catch.ac.uk/example-medical-clinical-academic-training-pathway). In the British medical context, doctors and dentists share the same pathway.

The diversity in the medical academic workforce is pivotal to increase innovation in healthcare, deliver high quality patient care, and provide visible role models and mentoring support for students and early career professionals, especially for those from minoritised ethnic backgrounds(5, 6). This also brings benefits to public health such as reducing health inequalities and building long term trustful relationship with disadvantaged communities (7, 8).

Various challenges have already been recognised for CA, such as limited funding, financial insecurity, limited CA positions, and the demanding workload of a clinical role (9-13). For CA from minoritised backgrounds, those challenges could be compounded. For example, for female CA, social expectations around family commitment and parenting responsibilities could result in their being unable to pursue CA careers (9, 14). In some cases, parental leave is limited by gender-based discrimination and sexism in the work culture (9). Barriers such as the under-

For peer review only - http://bmjopen.bmj.com/site/about/guidelines.xhtml

representation of ethnic minority colleagues in workplaces, a lack of cultural competency and trust from people at senior levels contribute to the challenges for CA from ethnic minority groups(13, 15).

Previous work has highlighted the value of intercalation for healthcare students to the diversity of the CA pipeline (11, 16). Interrupting professional training to develop the research skills in our early career future healthcare professionals is critical to widen access and participation for underrepresented groups in clinical academia. The removal of Educational Achievement scores from the allocation process for foundation posts is a cause for alarm, as it will have far-reaching consequences for student uptake of intercalation and amplify the inequities in the clinical academic pipeline (17). Significant investment is required to prevent further inequity at early career stages, such as funding and scholarships to students from disadvantaged backgrounds.

Increasing the diversity of the CA workforce is emphasised in policies internationally (18). In the UK, the NHS Race Equality Action Plan presented a mandatory workforce race equality standard (19 20). According to Kline et al. (21), in Northern England (which includes Greater Manchester) Ethnic minority representation at board and very senior management level was significantly lower than in the overall NHS workforce and also in the local communities served (21). Therefore, some regional actions have been taken to tackle the situation. The Manchester Foundation Trust introduced 'the Trust's Equality, Diversity and Inclusion Strategy 2019-2023' to ensure a representative and supported workforce (22). There has been an improvement in diversity of CA (gender and ethnicity) both in UK academic institutions and in the NHS (1, 23). Whilst there has been an improvement in the representation of CA, more work still needs to be done to attract and importantly sustain a diverse population of CA.

It is critical to understand the negative and positive experience of doctors and dentists from minoritised backgrounds, the barriers they experience and areas of support and institutional good practice in removing any systemic barriers. This data will inform recommendation at a local level which will also be applicable to a wider UK context.

For peer review only - http://bmjopen.bmj.com/site/about/guidelines.xhtml

METHODS

Design

A qualitative study using semi-structured interviews and focus groups was conducted to explore the experiences of the CA doctors and dentists in Greater Manchester, with a particular focus on those identifying as being from minoritised backgrounds (including but not limited to gender, race/ethnicity, and/or sexual orientation).

Setting and samples

Theoretical and snowball sampling were employed to recruit CA (doctors and dentists, in research-focused, teaching-focused, and research and teaching posts) and medical and dental students (years 3-5) from the University of Manchester, clinicians in Greater Manchester, key senior stakeholders and gatekeepers to CA and clinical posts in Greater Manchester. The aim of theoretical sampling is to "generate theory whereby the analyst jointly collects, codes and analyses his data and decides what data to collect next and where to find them in order to develop his theory as it emerges" (24). Snowball sampling is a technique where existing participants provide referrals of potential participants interested in taking part in the study (25). In order to capture the diversity of experiences, participants were recruited from a wide range of health settings, at various stages of their careers, and with an emphasis to include minoritised profiles.

Data collection

Potential participants were identified through the members of established medical education networks, clinical academic training and academic networks of the research team, and the snowball sampling approach involving already interviewed participants. Posters and social media platforms (such as Twitter and Faculty newsletters at the University of Manchester) were used to advertise the study. Any potential participants who were interested in taking part were able to contact the authors directly. All potential participants were provided with an online information sheet and gave fully informed consent before and during participation in the research. The research was designed to take into account the busy schedule of clinical academics, clinicians, and stakeholders, and the participants were given the option to choose between individual semi-structured interviews and focus groups. All participants, except the students, choose the individual interview because it was easier to fit in their schedule.

For peer review only

Interviews topic guides for semi-structured individual interviews and focus groups are available as supplemental files (Supplement 1).

Previous studies have shown Information power (26) to be important when the intention is to collect a rich account of experiences of participants. This means that the more information the sample holds (such as that provided with the in-depth interviews in the present study) the desired information power is achieved with fewer participants. The concept of information power focuses on how to accurately represent participants views, generate in-depth data, and cover the variability of relevant events (26).

Both focus group and individual interviews were carried out virtually through Zoom [5.13, Zoom Communications] or Microsoft Teams [1.5, Microsoft], lasting between 17 and 61 minutes. The interview topics included experiences of CA career pathway or stake holder perspective, barriers/facilitators encountered, especially for people from minoritised groups, and experiences of local research cultures. Brief field notes were taken for all interviews and focus groups to facilitate data analysis and identify the initial coding. All interviews and focus groups were conducted by first two authors (CL & CG). Data collection was undertaken from September 2022 to February 2023.

Data analysis

All interviews and focus groups were digitally recorded, transcribed and analysed following Braun and Clarke's reflexive approach to thematic analysis (27). Further, to consider existing qualitative research on the careers of CA and the barriers faced by minoritised groups, the interpretation of the results was integrated with the principle of 'reconstruction' from the extended case method proposed by Burawoy, in which anomalies in relation to an established theoretical corpus are considered and integrated in the analysis through the addition of new themes (28).

RESULTS

The study aimed to explore the experiences, barriers, and facilitators that CA (doctors and dentists) encounter in pursuing a CA career. A total of 43 participants took part in either an interview (37 clinical academic, clinician, and stakeholder participants) or a focus group for this study (6 medical or dental student participants). In this study, 21 men and 22 women with

For peer review only - http://bmjopen.bmj.com/site/about/guidelines.xhtml

a wide range of roles participated (19 clinical academics, 5 clinicians, 13 stakeholders and 6 medical or dental students). The 6 students were all in their 20s, the other participants were aged between 30 and 70 (9 in 30-39; 7 in 40-49; 18 in 50-59 and 3 in 60+). Of the 43 participants, 32 were White, 9 were Asian, and 2 were Black. The semi-structured interview framework focused on both the barriers and facilitators for minoritised groups and also general barriers and facilitators applicable to all CA.

Six main themes were generated: Theme 1: A highly competitive pathway that gives limited job security; Theme 2: Balancing two high-workload careers; Theme 3: Lack of representation and role models as limits to belonging; Theme 4: Being foreign or perceived as such; Theme 5: Childcare and gender inequalities; Theme 6: Support and recognition through mentorship. Of these themes, the first three describe barriers common to all CA, themes 3-5 describe barriers that impact in particular participants from minoritised groups, while theme 6 describes one of the main facilitating factors for all CA. Details of themes and sub-themes are summarised in table 1.

Table 1: themes and sub-themes

Theme	Sub-theme
A highly competitive pathway that gives limited job security	Financial challenges
	Jobs insecurity
	Limited funding
Balancing two high-workload careers	Heavy workload
	Limited time for conducting research
Lack of representation and role models as limits to belonging	Diverse role models
	Support from peers with a similar background
Being foreign or perceived as such	A feeling of belonging
	A language barrier
Childcare and gender inequalities	Maternity leave
	Parenting responsibility
Support and recognition through mentorship	Supportive mentorship from peers with similar experience
	Useful advice from mentors

For peer review only - http://bmjopen.bmj.com/site/about/guidelines.xhtml

Theme 1: A highly competitive pathway that gives limited job security

Participants expressed a strong feeling of insecurity in relation to their own career as CA, describing CA as a more precarious pathway when compared to a clinical career. Participants at all levels of seniority recognised that there is fear while holding fixed-term positions of losing one's jobs, and of being unable to control how long CA employment would be based on the success rate of obtaining grants and the duration of the grants.

Several participants mentioned the *financial challenges* of the CA pathway, including precarity and uncertainty linked to fixed-term contracts and *limited funding*. It was seen as problematic that CA needed to secure funding to continue in their career paths, particularly in the early stages of their career. One gatekeeper said:

> *"There is a lot of career uncertainty in academic life. I think it's becoming increasingly so since, say, in the last decade or so, because of funding difficulties in higher education. Determined junior members of staff may start out enthusiastically trying to cut a path in academic life, but I think they become frustrated at the lack of security in their job and it's all too easy to feel that you could move into a less rigorous discipline."* [G04]

Feelings that they were at risk of *losing their jobs* after the completion of the Academic Clinical Lecturer (ACL) training were widely reported by the participants. One said:

> *"I suppose the key step is if you're at the ACL level either with a formal ACL or trying to get your first independent fellowship, if that doesn't get funded by the time your ACL ends then just to earn a living you have to go back to the NHS, and then I think it's quite difficult. You've got to be very driven to be able to drive forward a fellowship application whilst you're still working full time in the clinical arena."* [G07]

One of the privileged routes to enter the CA pathway is through fellowships, and *funding opportunities* for fellowships are extremely competitive. One participant mentioned:

For peer review only - http://bmjopen.bmj.com/site/about/guidelines.xhtml

"There's a sort of a wish that everybody will go and get a very prestigious MRC [Medical Research Council] or Wellcome Trust fellowship, but actually there's not enough of those to go around." [G06]

The participants who obtained the most prestigious fellowships and had straightforward careers (e.g. transiting from specialisation to academic clinical fellow and then lecturer progressing on to a permanent CA position) also reported being concerned or anxious about finding their next job or securing their next grant in academia. The participants felt that continuous and secure employment was increasingly difficult to find. Therefore, uncertainty of subsequent employment and funding was also described as a major barrier to staying in the field. For example:

"As opposed to research, where you literally, if you don't have the next thing [research job]*, it's like where is your next pay cheque coming from. So, also making that not a thing for people who are specifically research. I don't know how you would do that. It's just, yeah. I know there's no easy answer to some of these things."* [CA07]

Whilst all participants were keen to pursue CA career pathways, the *uncertainty of employment* as a CA emerged as a constant source of anxiety. As a result, the participants felt that they needed to prioritise and maintain an income to meet their basic living costs. A number of participants believed that a purely clinical career was more attractive due to its stability and clearly defined clinical progression route. One participant also explained:

"Academia does not support a family life, whereas the NHS does so. And when I speak to my peers who are in academia, that's what they say: they are just not supported. And the way that academia works, is that you're going from contract to contract and grants, no stability at all. So, I'm glad I'm not an academic for that reason." [G08]

The importance of securing and *sustaining funding* was noted as a key challenge by a number of the participants. Supporting early and mid-career CA fellows through internal and external bridging funds whilst they are preparing for their next grant application was identified as a

For peer review only - http://bmjopen.bmj.com/site/about/guidelines.xhtml

facilitator for career progression. One mid-career clinical academic shared the following experience:

> "*These things* [funding] *are very competitive. I was very fortunate: I got the *** Clinical Training Fellowship, the first I applied for, and I was successful, so that was fortunate. The Intermediate Fellowship took me a few goes and at the time there was no... there were no schemes to support clinical academics in that intermediate phase. And I think it's a bit better now because there's the NIHR clinical lectureship scheme that provides some reaching* [bridging] *support, because you can't jump straight from a clinical train to a PhD and straight to an intermediate fellowship. So, I was very fortunate to be working in an environment where there was funding to support me, um, but that was a challenge."* [CA18]

Theme 2: Balancing two high-workload careers

Clinical academic careers can offer the benefits and rewards of making a difference to patients and having the space to pursue research projects. However, the challenge of how to *balance an academic role with a clinical career* was one of the common barriers mentioned:

> *"And I say to all clinicians who express an interest in academic work that everything is possible, but that you are embarking upon a career that has a clinical component and academic component, and you will be juggling, and it will affect the intensity of your work. So, people have the awareness that this isn't gonna be the easiest route."* [G11]

The workload for both the clinical and academic roles often goes beyond what is anticipated based on the clinical academic job plan. Effective line management and responsive workload management between both roles can reduce stress and improve satisfaction whilst meeting the demands of both roles. From participants' reports, *workloads* go beyond the official hours both in the clinic and in academia. In addition, balancing research, teaching, and clinical roles was a common challenge:

For peer review only - http://bmjopen.bmj.com/site/about/guidelines.xhtml

"Two-and-a-half days were taken up by my clinical work, which is the same as was, and two days were taken up for teaching, so it's not quite accurate to say I had no research time, but I had half a day of research time in that five days and when you are a clinical academic you need to do lots of things to build up your name, especially when you're starting. You need time to go for grants. You need time to write papers. You need time to network and do increasing amounts and gain momentum." [CA09]

A common difficulty was the feeling of being considered as not doing enough work in the clinic compared to full-time colleagues, with the research work in some cases being considered less strenuous than the clinical work that CA are expected to do in the same measure as full-time colleagues. For junior clinicians this further could mean being potentially seen as someone not acquiring the necessary clinical skills, as in the case of this junior clinical academic:

"If we are not in theatre learning, we won't acquire our skills. Particularly from a clinical perspective it requires a lot of flexibility from trainers. These are surgeons, they just live, breathe, eat surgery. I am there 50% of the time. Sometimes they see me, sometimes they don't, and then they ask me 'are you here tomorrow?' You say 'no, I am not here'. You feel inadequate, you feel like you are not giving 100%." [CA06]

Theme 3: Lack of representation and role models as limits to belonging

Barriers linked to systemic forms of discrimination can act on a more personal level, undermining a person's sense of self-worth and well-being. Sexism, racism, and other forms of discrimination that individuals experience throughout their life can impact their work life.

"But as an ethnic minority you feel those, you have grown up with them and some of them are not overt. Prejudices are, for me, I can usually sense them rather than someone [...] *overtly saying no* [...] *You have heard it so many times, you kind of just think, right, okay, I am going to go and knock on a different door. I would say those things are the things that made me constantly more think I have to prove myself, I have to prove myself."* [CA06]

For peer review only - http://bmjopen.bmj.com/site/about/guidelines.xhtml

The importance of representation and a feeling of belonging is crucial for a diverse and dynamic workforce. However, it is common for some specialties, research fields, and/or senior leadership positions to be occupied by white British men. Many of the women participants have for example described leadership positions within academia as closed circles. Indeed, many of the women who did not think that their gender has negatively impacted on their career recognise that some higher roles are much more male-dominated.

> *"We still live in a world where they* [men] *dominate senior academic and senior clinical and senior college and senior government type positions, and so they make a lot of decisions. I think that they occupy that space more than women, and that is levelling up all the time. I don't think they've ever stood in my way."* [CA11]

The lack of *diverse role models* and peers with whom it was possible to identify was discussed, along with the feeling of being out of place in different settings.

> *"If I go to a meeting of researchers* […] *I might be the only South Asian in the room. That's when I feel like a minority; I never feel like a minority in clinical meetings."* [CA09]

> *"And even less so when you're black and woman, there's even less of that* [ethnic minority women in CA] *And sometimes it's like, oh…can you do this?"* [CA07]

Greater Manchester is arguably one of the most diverse areas of the UK. The importance of diversifying the CA pipeline was recognised in the Trust's Equality, Diversity, and Inclusion Strategy 2019-2023 (introduced by Manchester University NHS Foundation Trust) (22) which led to actions such as improving diversity of interview panels. Some interviewees reported positive experiences, such as this female ethnic minority CA:

> *So, I went and did the interview and my interview panel actually was a group of all women, all different ethnicities and, I don't know, immediately I just felt way more at ease with them. I felt like I could be myself. We spent 45 minutes talking about me and*

For peer review only - http://bmjopen.bmj.com/site/about/guidelines.xhtml

my career goals and what I wanted and they really…I felt like they really got to know me and they gave me that time and respect to open up and answer questions. [CA04]

Theme 4: Being foreign or perceived as such

Along with the difficulties linked to race and ethnicity, some of the barriers discussed by the interviewees were more specifically linked to not being British-born or not being British citizens. Having a foreign accent was recognised as a marker of not being British-born, and potentially non-British-trained. As a result, CA with the same ethnicity could have very different experiences. As one participant discusses:

"The other thing I think is something to do with how well you can speak and whether you have an unusual accent, which some people do, sometimes that accent can be taken as a bias against subconsciously, I feel. It hasn't happened to me, but I have seen other people who are very capable, but with a strong accent […] *maybe could have done better."* [C04]

Language barriers, even if very modest, were also recognised as potentially creating a disadvantage in publishing and therefore building esteem and an academic career. Others underlined the role that discrimination can play, such as the peer review of grants and articles. Interviewees have underlined how in these cases the doubt remains whether a non-English name might negatively influence the outcome.

Theme 5: Childcare and gender inequalities

The most discussed barriers experienced along gender lines were linked to maternity and childcare and return to work following a period of maternity leave was described as difficult by a number of interviewees, particularly where the expectation was that work can resume without any disruptions.

"The one thing [a supervisor] *said to me was 'you just need to make sure that your childcare is bomb-proof'. So, I don't know how you can manage to have childcare that is completely perfect."* [G11]

t

For peer review only - http://bmjopen.bmj.com/site/about/guidelines.xhtml

Although maternity leave is recognised when accounting for career breaks in the academic CV, a number of interviewees felt that interruptions for maternity were not taken into account when considering the track record of candidates for promotion and career progression. *Maternity* was recognised as a source of discrimination even by women who did not have children. One stakeholder observed:

> *"I think [that] for women [it is difficult] to progress because of the time barrier. If you're then having children and you're trying to juggle a clinical academic career, it's almost like you have two full time jobs… So, we lose a lot of women, and that's why you tend to see a lot of male clinicians who are then on the research path."* [G12]

Childcare was further linked by several participants to barriers for the CA careers. A number of interviewees discussed how childcare continues to largely fall upon women, and how it is difficult to reconcile the already high workload of clinic, research and/or teaching with parenting. The difficulty of managing the different workloads combined with the demands in terms of flexibility and potential need to relocate linked to the academic career. As a result, a number of interviewees observed how a purely clinical career was more attractive as it was more stable with less workload outside hospital hours, and how this might be an incentive to abandon the CA pathway.

Theme 6: Support and recognition through mentorship

Mentorship was identified as a main facilitator for CA career development. Mentorship relationships were mostly built through personal contacts, professional networks, or formal mentorship programs. Typically, the participants were more likely to seek mentorship from people who have the same or similar life experiences and were successful in CA. The participants believed that supportive mentorship could facilitate exploring their research interests, building their portfolio and expanding their professional networks. Mentors were able to provide deep insight into CA career pathways, for example:

> *"The person who's helped me the most was my PhD supervisor. He is a clinical academic; he interviewed me the first time round and didn't give me the fellowship, but*

For peer review only - http://bmjopen.bmj.com/site/about/guidelines.xhtml

encouraged me to seek other opportunities. He knew… because he'd been through it himself, he was aware of the trajectory, he was aware of what was needed, he really helped me in terms of how to write a fellowship application, how to sell yourself, really, which I think is something that I struggle with. So he's been very instrumental in helping my career, definitely, and seeing that I potentially have a future in this." [CA03]

Participants who self-identified as earlier career researchers, pointed out the importance of *senior and peer mentors* in CA career pathways. Beyond the academic support, some participants also noted that mentors are pivotal when they encounter challenges in the field. The benefits of mental or emotional support from these mentors were reported. Based on their advanced knowledge or experience, mentors were in a good position to offer *advice on CA career pathways*. The participants felt they were protected: in a safe place. They believed these mentors could help them to develop into professional and independent CA, for example:

"I had a fantastic mentor […] *and she kind of took me under her wing. She could see I felt a bit lost. I didn't get the training number. I was, you know, five hours away from my family and she kind of just gave me this motivation. And some focus to say, 'Right. You're next. I know it's difficult for you up here and you're lonely, but you need to focus on the next step.' And she was pivotal really, in that she really believed in me. Like, really, for somebody, I didn't know very well. I don't know. She had this incredible ability to make me quite confident in my skills."* [CA06]

DISCUSSION

The participants to our research project form a diverse group including CA, clinicians, students and stakeholders, which has made it possible to understand a wide range of difficulties and problems impacting decision-making and career choices. Six themes have been identified, with two describing general barriers experienced by CA, three capturing further the complex experiences of minoritised groups, and one identifying an important facilitator.

This project has corroborated previous research (9, 10) in showing the difficulties in managing two demanding careers and in finding a balance for activities with a significant workload that

For peer review only - http://bmjopen.bmj.com/site/about/guidelines.xhtml

are difficult to scale to part-time positions, and how this creates challenges for career advancement, especially for people with childcare responsibilities.

Both this study and previous research reported that various challenges such as job insecurity and heavy workload could be key barriers to pursuing a clinical academic career (9-13). Several new elements emerge from the analysis of our data. Among the general barriers, our participants mentioned recent changes in the career pathway, including the reduction of available positions, coupled with the expectation of obtaining prestigious fellowships before advancing in CA careers.

The participants highlighted the degree to which obtaining such fellowships requires time, support from experienced colleagues, and availability of bridge funding to allow time to re-apply in case of rejection. While available in the context studied, such resources are unequally distributed, and require in turn a significant time and financial investment for an uncertain outcome. In particular, support from peers and colleagues, particularly at a senior level, were recognised as a key facilitator to tackle these challenges, both in our study and in previous literature (10, 11). A recent systematic review of the existing interventions to support clinical academic careers for doctors and dentists found that protected research time could enhance CA retention and career progression (11). In addition, to bolster the feeling of belonging for minoritised CA, representation and inclusion is crucial.

One of the main individual barriers are the ways in which being minoritised hinders one's confidence. Ethnic minority participants reported episodes of racism, in their careers, but further discussed how experiences of discrimination and racism suffered in other spheres of life can have an impact also on their self-worth in a professional setting. A strong sense of being an "outlier" is still commonly reported from women and minority populations in the research environment in the literature (9,13-15). This might explain the feeling of unbelonging that further erodes confidence. For example, women participants perceived men occupying the majority of the senior and management positions as disempowering. Despite improvement in female representation in leadership positions, gender imbalance can impact career progression through the phenomena already identified as glass ceiling and/or sticky floors (9, 10). Ethnic minority participants have reported similar experiences, with further issues such as having a

For peer review only - http://bmjopen.bmj.com/site/about/guidelines.xhtml

name perceived as foreign and being potentially disadvantaged in being evaluated for publications and grants.

Taken together these data highlight the range of systemic barriers experienced by minoritised CA. These ranged from how having an accent was perceived as limiting progression, to lack of role models, difficulties in balancing childcare commitments and two highly demanding roles. The study also highlighted key enablers through bridging financial support, grant writing support and recognising lived experience and supporting career progression through mentorship. These findings will help inform individuals, senior leaders and organisations of the facilitators and barriers that impact on CA career progression and help policy development for those leading on clinical academic training programmes and delivery of a targeted equality and diversity strategy.

There are limitations to this study: the fact that only a quarter of the respondents were from minoritised groups should be taken into account in the analysis of the results. Further studies with wider and more diverse samples might be helpful to enhance transferability in qualitative research. Meanwhile, as one aim of this study was to understand the experiences of minoritised groups, the composition of the research team should also be considered. Our team is diverse in terms of ethnicity and national origin, but is entirely female, which can have had a role in the collection and analysis of the data.

CONCLUSIONS

The study showed that there are several barriers that current and prospective CA face. It has also shown that barriers and difficulties are increased for participants from minoritised groups. To remove these barriers, cultural and structural interventions are needed. On the one hand, it is important to identify forms of financial support for early career CA, in order to make the CA pathway more financially stable. On the other hand, it is important to encourage wider social and cultural changes that can reduce the feelings of unbelonging that women and ethnic minority participants in particular have expressed. These findings and recommendations are of relevance for senior leaders and organisations involved in clinical academic training and more general in equality & diversity and careers strategy.

For peer review only - http://bmjopen.bmj.com/site/about/guidelines.xhtml

Acknowledgments

Thanks to Prof Colette Fagan and Prof Adam Danquah, University of Manchester for helpful discussions during the course of this project. Thanks to clinicians, clinical academics, students and stakeholders who participated or facilitated this study.

Contributors

HR and NG conceived of and led the study as principal investigators; CL and CG conducted data collection and data analysis; GF, HR and NG contributed qualitative data analysis. All authors (CL, CG, HR, GF, RC and NG) made contributions to the manuscript and reviewed the final submission.

Funding: This work was supported by University of Manchester and Wellcome matched funding reference 204796/Z/16/Z ISSF - Wellcome ISSF 3 - EDI.

Declaration of Interest: None

Patient and public involvement

Patients and/or the public were not involved in the design, or conduct, or reporting, or dissemination plans of this research.

Patient consent for publication

Not applicable.

Ethics approval

This study involves human participants and was approved by the University of Manchester Ethics Committee (Reference: 2022-13791-23819). Informed consent was obtained from all participants in the study before taking part.

Data availability statement

The interview and focus group data used in this study are not publicly available due to integrity statements in the ethics approvals.

For peer review only

For peer review only - http://bmjopen.bmj.com/site/about/guidelines.xhtml

REFERENCES

1. Medical Schools Council. Clinical academic survey [Available from: https://www.medschools.ac.uk/clinical-academic-survey accessed 10/04 2023.
2. Nicole W. Survey of Medical Clinical Academic Staffing Levels London: Medical Schools Council; 2018 [Available from: https://www.medschools.ac.uk/media/2491/msc-clinical-academic-survey-report-2018.pdf2023.
3. Linden JA, Baird J, Madsen TE, et al. Diversity of leadership in academic emergency medicine: Are we making progress? *The American Journal of Emergency Medicine* 2022;57:6-13. doi: https://doi.org/10.1016/j.ajem.2022.04.009
4. Richter KP, Clark L, Wick JA, et al. Women physicians and promotion in academic medicine. *New England Journal of Medicine* 2020;383(22):2148-57. doi: 10.1056/NEJMsa1916935
5. Parker RB, Stack SJ, Schneider SM. Why diversity and inclusion are critical to the American college of emergency physicians' future success. *Annals of Emergency Medicine* 2017;69(6):714-17. doi: 10.1016/j.annemergmed.2016.11.030 [published Online First: 2017/02/01]
6. Alsan M, Garrick O, Graziani G. Does diversity matter for health? Experimental evidence from Oakland. *American Economic Review* 2019;109(12):4071-111. doi: 10.1257/aer.20181446
7. Beebeejaun K, Littleford K. A diverse public health workforce is more important than ever. *BMJ (Clinical research ed)* 2023;380:p447. doi: 10.1136/bmj.p447
8. Marmot M. Building back fairer in Greater Manchester and the country. Royal Society Open Science 2021;8(10):211454. doi: doi:10.1098/rsos.211454
9. Ranieri VF, Barratt H, Rees G, et al. A qualitative study of the influences on clinical academic physicians' postdoctoral career decision making. *Academic Medicine* 2018;93(11):1686-93. doi: 10.1097/acm.0000000000002141 [published Online First: 2018/01/26]
10. Trusson D, Rowley E, Bramley L. A mixed-methods study of challenges and benefits of clinical academic careers for nurses, midwives and allied health professionals. *BMJ Open* 2019;9(10):e030595. doi: 10.1136/bmjopen-2019-030595
11. Raine G, Evans C, Uphoff EP, et al. Strengthening the clinical academic pathway: a systematic review of interventions to support clinical academic careers for doctors and dentists. *BMJ Open* 2022;12(9):e060281. doi: 10.1136/bmjopen-2021-060281
12. Kehoe A, Crampton P, Buchanan J, et al. Tips to Support the Recruitment, Retention, and Progression of Clinical Academics. *Medical Science Educator* 2022;32(2):503-09. doi: 10.1007/s40670-022-01512-1
13. Finn G, Morgan J. From the sticky floor to the glass ceiling and everything in between: A systematic review and qualitative study focusing on gender inequalities in Clinical Academic careers, 2020.
14. Vassie C, Smith S, Leedham-Green K. Factors impacting on retention, success and equitable participation in clinical academic careers: a scoping review and meta-thematic synthesis. *BMJ Open* 2020;10(3):e033480. doi: 10.1136/bmjopen-2019-033480
15. Woolf K, Rich A, Viney R, et al. Perceived causes of differential attainment in UK postgraduate medical training: a national qualitative study. *BMJ Open* 2016;6(11):e013429. doi: 10.1136/bmjopen-2016-013429
16. Finn GM, Crampton P, Buchanan JA, et al. The impact of the COVID-19 pandemic on the research activity and working experience of clinical academics, with a focus on gender

For peer review only - http://bmjopen.bmj.com/site/about/guidelines.xhtml

and ethnicity: a qualitative study in the UK. *BMJ Open* 2022;12(6):e057655. doi: 10.1136/bmjopen-2021-057655
17. Finn GM, Crampton PES, Kehoe A, et al. Will removing intercalation undermine our clinical academic foundations? *BMJ (Clinical research ed)* 2021;374:n1527. doi: 10.1136/bmj.n1527
18. Boatright DH, Samuels EA, Cramer L, et al. Association between the Liaison Committee on Medical Education's Ddversity standards and changes in percentage of medical student sex, race, and ethnicity. *Jama* 2018;320(21):2267-69. doi: 10.1001/jama.2018.13705 [published Online First: 2018/12/05]
19. Priest N, Esmail A, Kline R, et al. Promoting equality for ethnic minority NHS staff—what works? *BMJ : British Medical Journal* 2015;351:h3297. doi: 10.1136/bmj.h3297
20. Ovseiko PV, Taylor M, Gilligan RE, et al. Effect of Athena SWAN funding incentives on women's research leadership. *BMJ (Clinical research ed)* 2020;371:m3975. doi: 10.1136/bmj.m3975
21. Kline R, Naqvi H, Razaq SA, et al. NHS Workforce Race Equality Standard: 2016 Data Analysis Report for NHS Trusts 2017 [Available from: https://www.england.nhs.uk/wp-content/uploads/2017/03/workforce-race-equality-standard-data-report-2016.pdf accessed 20 May 2023.
22. Equality, Diversity and Inclusion: Manchester Foundation Trust; 2023 [Available from: https://mft.nhs.uk/the-trust/equality-diversity-and-inclusion/ accessed 10/04 2023.
23. NHS Workforce Race Equality Standard 2022 [Available from: https://www.england.nhs.uk/wp-content/uploads/2022/04/Workforce-Race-Equality-Standard-report-2021-.pdf accessed 10/04 2023.
24. Glaser BG, Strauss AL. *The Discovery of Grounded Theory: Strategies for Qualitative Research*. 2017 Routledge.
25. Parker C, Scott S, Geddes A. . Snowball Sampling, In P Atkinson, S Delamontet al. (Eds.), *SAGE Research Methods Foundations*. 2019. https://doi.org/10.4135/9781526421036831710
26. Malterud K, Siersma VD, Guassora AD. Sample Size in Qualitative Interview Studies: Guided by Information Power. *Qualitative Health Research* 2016;26(13):1753-1760. doi: 10.1177/1049732315617444
27. Braun V, Clarke V. Reflecting on reflexive thematic analysis. *Qualitative Research in Sport, Exercise and Health* 2019;11(4):589-97. doi: 10.1080/2159676X.2019.1628806
28. Burawoy, M. The Extended Case Method. *Sociological Theory* 1998;16(1): 4-33. doi: 10.1111/0735-2751.00040

For peer review only

Supplement 1:

Indicative interview question stems – SCALE

Please note, the following are suggested, iterative question stems. The group interviews and individual interviews will both be semi-structured in nature, so interviewers will also be able to follow interesting lines of questioning/prompt further than the lists supplied below, if necessary. Interviewers will meet regularly to discuss interesting insights from the interviews, and will have the agency and ability to modify, evolve or add questions as appropriate, and as befits the iterative design of this qualitative research

Clinician academics focus groups

1. Tell us about your journey into clinical academia (Icebreaker question).
2. What facilitated your journey?
    a. (If appropriate): Why do you think you experienced such facilitators?
3. Did you experience any difficulties or barriers to your career?
    a. If so, please elaborate.
    b. Why do you think you experienced these barriers?
4. What is the research culture of your academic institution like?
5. Has the culture you've experienced at your academic institution influenced any decisions you've made about your career?
    a. If so, how?
6. Has the culture you've experienced within medicine/dentistry and academia more generally influenced any decisions you've made about your career?
    a. If so, how?
7. Thinking about any barriers or negative experiences of culture that have been mentioned, what changes could be made on an institutional level to enable clinical academics from minoritised backgrounds to flourish?
8. Is there anything else you wish to tell us about your experiences of clinical academia within Greater Manchester?

Clinicians focus groups

1. Tell us about your journey into clinical practice to date (Icebreaker question).
2. What facilitated your journey?
    a. (If appropriate): Why do you think you experienced such facilitators?
3. Did you experience any difficulties or barriers to your career?
    a. If so, please elaborate.
    b. Why do you think you experienced these barriers?
4. How do you view careers in clinical academia?
    a. Does anyone have experience as a clinical academic? If you left an academic post, can you tell us why?
    b. Have you ever considered a clinical academic career? If the answer is yes but you chose not to pursue a clinical academic career, can you tell us why?
    c. For those that haven't considered clinical academic careers, can you tell us why? Did anything in particular put you off?

For peer review only

5. In Greater Manchester more specifically, do you get any sense of what it's like to be a clinical academic?
    a. What do you think about the culture surrounding research in Greater Manchester?
    b. Have your experiences or perceptions of this culture influenced the decisions you've made about your career in any ways?
6. Thinking about any barriers or negative experiences of culture that have been mentioned, what changes could be made on an institutional level to enable clinicians from minoritised backgrounds to pursue clinical academic careers, if they wish?
7. Is there anything else you wish to tell us about your experiences of the research culture or academia within Greater Manchester?

Medical and dental students focus groups

1. How familiar are you with the clinical academic career pathway? (Icebreaker question).
2. What kind of information, if any, have you received about clinical academic career during your studies?
3. Would you be interested in pursuing an academic career along with your clinical one?
4. Do you have an opinion about intercalated degrees?
5. Do you see difficulties or barriers to start a clinical academic career?
    a. If so, please elaborate.
    b. What do you think is the reason behind these barriers?
6. What is the institutional culture of the University of Manchester like?
7. Has the culture you've experienced at the university influenced any plans you've made about your career?
    a. If so, how?
8. Thinking about any barriers or negative experiences of culture that have been mentioned, what changes could be made on an institutional level to enable people from minoritised backgrounds to start a clinical academic career?
9. Is there anything else you wish to tell us about your experiences within the University of Manchester?

Gatekeeper interviews

1. Can you tell me about your current role? (Icebreaker question)
    a. Do you have/ What responsibilities do you have in terms of managing clinicians/clinical academics?
    b. Do you have/ What responsibilities do you have in terms of making selection decisions in clinical academia?
2. What have your experiences of managing clinicians/clinical academics and/or making selection decisions been like?
3. From your perspective, what are the facilitating factors for clinicians looking to build clinical academic careers?
4. From your perspective, what are the barriers for clinicians looking to build clinical academic careers?

For peer review only - http://bmjopen.bmj.com/site/about/guidelines.xhtml

5. Do you feel able to support the staff you manage to confront or tackle the barriers they may experience?
    a. If yes, can you give some examples of what this support looks like in practice? What resources does this support require?
    b. If no, can you please explain why?
6. If you have experience making selection decisions, do you feel able to confront any barriers to clinical academic careers in your role within selection?
    a. If yes, how?
    b. If no, why not? Could anything change to enable you to challenge the barriers you are aware of?
7. Thinking specifically about the experiences of those you manage who are from minoritised backgrounds, are there any additional facilitating factors for minoritised clinicians looking to build clinical academic careers?
8. Thinking specifically about the experiences of those you manage who are from minoritised backgrounds, are there any additional barriers for minoritised clinicians looking to build clinical academic careers?
9. Do you feel able to support the staff you manage from minoritised backgrounds to confront or tackle the barriers they may experience?
    a. If yes, can you give some examples of what this support looks like in practice? What resources does this support require?
    b. If no, can you please explain why?

For peer review only

Standards for Reporting Qualitative Research (SRQR)*

http://www.equator-network.org/reporting-guidelines/srqr/

Title and abstract

	Page/line no(s).
**Title** - Concise description of the nature and topic of the study Identifying the study as qualitative or indicating the approach (e.g., ethnography, grounded theory) or data collection methods (e.g., interview, focus group) is recommended	Page 1
**Abstract** - Summary of key elements of the study using the abstract format of the intended publication; typically includes background, purpose, methods, results, and conclusions	Page 1

Introduction

**Problem formulation** - Description and significance of the problem/phenomenon studied; review of relevant theory and empirical work; problem statement	Page 3
**Purpose or research question** - Purpose of the study and specific objectives or questions	Page 4

Methods

**Qualitative approach and research paradigm** - Qualitative approach (e.g., ethnography, grounded theory, case study, phenomenology, narrative research) and guiding theory if appropriate; identifying the research paradigm (e.g., postpositivist, constructivist/ interpretivist) is also recommended; rationale**	Page 4-5
**Researcher characteristics and reflexivity** - Researchers' characteristics that may influence the research, including personal attributes, qualifications/experience, relationship with participants, assumptions, and/or presuppositions; potential or actual interaction between researchers' characteristics and the research questions, approach, methods, results, and/or transferability	Page 15
**Context** - Setting/site and salient contextual factors; rationale**	Page 4-5
**Sampling strategy** - How and why research participants, documents, or events were selected; criteria for deciding when no further sampling was necessary (e.g., sampling saturation); rationale**	Page 4-5
**Ethical issues pertaining to human subjects** - Documentation of approval by an appropriate ethics review board and participant consent, or explanation for lack thereof; other confidentiality and data security issues	Page 16
**Data collection methods** - Types of data collected; details of data collection procedures including (as appropriate) start and stop dates of data collection and analysis, iterative process, triangulation of sources/methods, and modification of procedures in response to evolving study findings; rationale**	Page5

For peer review only - http://bmjopen.bmj.com/site/about/guidelines.xhtml
For peer review only

**Data collection instruments and technologies** - Description of instruments (e.g., interview guides, questionnaires) and devices (e.g., audio recorders) used for data collection; if/how the instrument(s) changed over the course of the study	Page 5
**Units of study** - Number and relevant characteristics of participants, documents, or events included in the study; level of participation (could be reported in results)	Page 6
**Data processing** - Methods for processing data prior to and during analysis, including transcription, data entry, data management and security, verification of data integrity, data coding, and anonymization/de-identification of excerpts	Page 5
**Data analysis** - Process by which inferences, themes, etc., were identified and developed, including the researchers involved in data analysis; usually references a specific paradigm or approach; rationale**	Page 6
**Techniques to enhance trustworthiness** - Techniques to enhance trustworthiness and credibility of data analysis (e.g., member checking, audit trail, triangulation); rationale**	Page 5

Results/findings

**Synthesis and interpretation** - Main findings (e.g., interpretations, inferences, and themes); might include development of a theory or model, or integration with prior research or theory	Page 6
**Links to empirical data** - Evidence (e.g., quotes, field notes, text excerpts, photographs) to substantiate analytic findings	Page 6-14

Discussion

**Integration with prior work, implications, transferability, and contribution(s) to the field -** Short summary of main findings; explanation of how findings and conclusions connect to, support, elaborate on, or challenge conclusions of earlier scholarship; discussion of scope of application/generalizability; identification of unique contribution(s) to scholarship in a discipline or field	Pag 14-15
**Limitations** - Trustworthiness and limitations of findings	Page 15

Other

**Conflicts of interest** - Potential sources of influence or perceived influence on study conduct and conclusions; how these were managed	Page 17
**Funding** - Sources of funding and other support; role of funders in data collection, interpretation, and reporting	Page 17

*The authors created the SRQR by searching the literature to identify guidelines, reporting standards, and critical appraisal criteria for qualitative research; reviewing the reference lists of retrieved sources; and contacting experts to gain feedback. The SRQR aims to improve the transparency of all aspects of qualitative research by providing clear standards for reporting qualitative research.

For peer review only - http://bmjopen.bmj.com/site/about/guidelines.xhtml

**The rationale should briefly discuss the justification for choosing that theory, approach, method, or technique rather than other options available, the assumptions and limitations implicit in those choices, and how those choices influence study conclusions and transferability. As appropriate, the rationale for several items might be discussed together.

Reference:

O'Brien BC, Harris IB, Beckman TJ, Reed DA, Cook DA. **Standards for reporting qualitative research: a synthesis of recommendations.** *Academic Medicine*, Vol. 89, No. 9 / Sept 2014 DOI: 10.1097/ACM.0000000000000388

For peer review only

For peer review only - http://bmjopen.bmj.com/site/about/guidelines.xhtml

Experiences of the clinical academic pathway: A qualitative study in Greater Manchester to improve the opportunities of minoritised clinical academics.

Journal:	*BMJ Open*
Manuscript ID	bmjopen-2023-079759.R2
Article Type:	Original research
Date Submitted by the Author:	19-Jan-2024
Complete List of Authors:	Lin, Chiu-Yi; The University of Manchester, Division of Pharmacy and Optometry, School of Health Sciences, Faculty of Biology, Medicine and Health<br>Greco, Cinzia; The University of Manchester, Centre for the History of Science, Technology and Medicine; The University of Manchester, Division of Medical Education, School of Medical Sciences, Faculty of Biology, Medicine and Health<br>Radhakrishnan, Hema; The University of Manchester, Division of Pharmacy and Optometry, School of Health Sciences, Faculty of Biology, Medicine and Health<br>Finn, Gabrielle; The University of Manchester, Division of Medical Education, School of Medical Sciences, Faculty of Biology, Medicine and Health<br>Cowen, Rachel; The University of Manchester, Division of Medical Education, School of Medical Sciences, Faculty of Biology, Medicine and Health<br>Gardiner, Natalie; The University of Manchester, Division of Diabetes, Endocrinology & Gastroenterology, School of Medical Sciences, Faculty of Biology, Medicine and Health
**Primary Subject Heading**:	Medical education and training
Secondary Subject Heading:	Qualitative research
Keywords:	MEDICAL EDUCATION & TRAINING, QUALITATIVE RESEARCH, Sexual and Gender Minorities

SCHOLARONE™
Manuscripts

I, the Submitting Author has the right to grant and does grant on behalf of all authors of the Work (as defined in the below author licence), an exclusive licence and/or a non-exclusive licence for contributions from authors who are: i) UK Crown employees; ii) where BMJ has agreed a CC-BY licence shall apply, and/or iii) in accordance with the terms applicable for US Federal Government officers or employees acting as part of their official duties; on a worldwide, perpetual, irrevocable, royalty-free basis to BMJ Publishing Group Ltd ("BMJ") its licensees and where the relevant Journal is co-owned by BMJ to the co-owners of the Journal, to publish the Work in this journal and any other BMJ products and to exploit all rights, as set out in our licence.

The Submitting Author accepts and understands that any supply made under these terms is made by BMJ to the Submitting Author unless you are acting as an employee on behalf of your employer or a postgraduate student of an affiliated institution which is paying any applicable article publishing charge ("APC") for Open Access articles. Where the Submitting Author wishes to make the Work available on an Open Access basis (and intends to pay the relevant APC), the terms of reuse of such Open Access shall be governed by a Creative Commons licence – details of these licences and which Creative Commons licence will apply to this Work are set out in our licence referred to above.

Other than as permitted in any relevant BMJ Author's Self Archiving Policies, I confirm this Work has not been accepted for publication elsewhere, is not being considered for publication elsewhere and does not duplicate material already published. I confirm all authors consent to publication of this Work and authorise the granting of this licence.

For peer review only

Experiences of the clinical academic pathway: A qualitative study in Greater Manchester to improve the opportunities of minoritised clinical academics.

Chiu-Yi Lin[1], Cinzia Greco[2,3], Hema Radhakrishnan[1], Gabrielle M Finn[3], Rachel L Cowen[3] and Natalie J Gardiner[4]

[1] *Division of Pharmacy and Optometry, School of Health Sciences, Faculty of Biology, Medicine and Health, University of Manchester, Manchester, M13 9PL, United Kingdom*

[2] *Centre for the History of Science, Technology and Medicine, University of Manchester, Manchester, M13 9PL, United Kingdom*

[3] *Division of Medical Education, School of Medical Sciences, Faculty of Biology, Medicine and Health, The University of Manchester, Manchester, M13 9PL, United Kingdom*

[4] *Division of Diabetes, Endocrinology & Gastroenterology, School of Medical Sciences, Faculty of Biology, Medicine and Health, University of Manchester, University of Manchester, Manchester, M13 9PL, United Kingdom*

Correspondence to Dr Hema Radhakrishnan
Carys Bannister Building,
University of Manchester,
Manchester M13 9PL
Email: Hema.Radhakrishnan@manchester.ac.uk
Tel: (+44)1613068763
Fax: (+44) 161 306 3969

Keyword: Medical education & training; Sexual and Gender Minorities; Qualitative Research

Word count: 5255

For peer review only

For peer review only - http://bmjopen.bmj.com/site/about/guidelines.xhtml

ABSTRACT

Objectives: The aim of this study was to explore the barriers and facilitators faced by clinical academics in the Greater Manchester region, with particular attention to the experiences of minoritised groups.

Design: A qualitative study using semi-structured interviews and focus groups was conducted. A reflexive thematic analysis was applied to identify key themes.

Setting: University of Manchester and NHS Trusts in the Greater Manchester region.

Participants: The sample of this study was composed of 43 participants, including clinical academics, stakeholders, clinicians, and medical and dental students.

Results: Six themes were identified. Clinical academics face several barriers and facilitators, some of which – 1) funding insecurity and 2) high workload between the clinic and academia – are common to all the clinical academics. Other barriers, including 3) discrimination that translates into struggles with self-worth and feeling of not belonging, 4) being or being perceived as foreign; and 5) unequal distribution of care duties, particularly affect people from minoritised groups. In contrast, 6) mentorship was commonly identified as one of the most important facilitators.

Conclusions: Cultural and structural interventions are needed, such as introducing financial support for early career clinical academics and intercalating health care students to promote wider social and cultural change and increase the feelings of belonging and representation across the entire clinical academic pipeline.

For peer review only - http://bmjopen.bmj.com/site/about/guidelines.xhtml

STRENGTHS AND LIMITATIONS OF THIS STUDY

1. In-depth interviews with a broad range of participants were conducted to explore the multidimensional barriers faced by clinical academics and the role played by stakeholders in supporting them.

2. An innovative qualitative analysis combining thematic analysis and Extended Case Method was used to analyse and interpret the data.

3. Only doctors and dentists were interviewed, professionals from other allied health disciplines were excluded.

4. The data focus only on the experiences of professionals working in Greater Manchester.

For peer review only

For peer review only - http://bmjopen.bmj.com/site/about/guidelines.xhtml

INTRODUCTION

The diversity profile of clinicians who choose the clinical academic (CA) pathway is not representative of the demographics of either clinical professionals with similar entry qualifications or that of the general population that they serve. Furthermore, the CA data shows that there are systematic inequalities within CA careers as the level of seniority increases (1). It is evident that the proportion of CA from minoritised backgrounds is still relatively low in the field. A survey in the UK found that only 33% of CA are female, ranging from only 13% to 58% in different specialities (2). This also largely reflects women often having lower academic rank and few attaining a leadership role in clinical academia (3, 4). In terms of ethnic profiles, minoritised ethnic CA are even less represented: only 17% of CA identify as having Black, Asian or Minority Ethnic backgrounds (2).

A clinical academic is a qualified healthcare professional who is engaged with clinical responsibilities in a healthcare setting whilst simultaneously holding an academic position and contributing to either research and teaching, or both. There is a well-defined career pathway for clinical academics with intercalation, based on clinical experience and academic qualification (https://www.catch.ac.uk/example-medical-clinical-academic-training-pathway). In the British medical context, doctors and dentists share the same pathway.

The diversity in the medical academic workforce is pivotal to increase innovation in healthcare, deliver high quality patient care, and provide visible role models and mentoring support for students and early career professionals, especially for those from minoritised ethnic backgrounds(5, 6). This also brings benefits to public health such as reducing health inequalities and building long term trustful relationship with disadvantaged communities (7, 8).

Various challenges have already been recognised for CA, such as limited funding, financial insecurity, limited CA positions, and the demanding workload of a clinical role (9-13). For CA from minoritised backgrounds, those challenges could be compounded. For example, for female CA, social expectations around family commitment and parenting responsibilities could result in their being unable to pursue CA careers (9, 14). In some cases, parental leave is limited by gender-based discrimination and sexism in the work culture (9). Barriers such as the under-

For peer review only

representation of ethnic minority colleagues in workplaces, a lack of cultural competency and trust from people at senior levels contribute to the challenges for CA from ethnic minority groups(13, 15).

Previous work has highlighted the value of intercalation for healthcare students to the diversity of the CA pipeline (11, 16). Interrupting professional training to develop the research skills in our early career future healthcare professionals is critical to widen access and participation for underrepresented groups in clinical academia. The removal of Educational Achievement scores from the allocation process for foundation posts is a cause for alarm, as it will have far-reaching consequences for student uptake of intercalation and amplify the inequities in the clinical academic pipeline (17). Significant investment is required to prevent further inequity at early career stages, such as funding and scholarships to students from disadvantaged backgrounds.

Increasing the diversity of the CA workforce is emphasised in policies internationally (18). In the UK, the NHS Race Equality Action Plan presented a mandatory workforce race equality standard (19 20). According to Kline et al. (21), in Northern England (which includes Greater Manchester) Ethnic minority representation at board and very senior management level was significantly lower than in the overall NHS workforce and also in the local communities served (21). Therefore, some regional actions have been taken to tackle the situation. The Manchester Foundation Trust introduced 'the Trust's Equality, Diversity and Inclusion Strategy 2019-2023' to ensure a representative and supported workforce (22). There has been an improvement in diversity of CA (gender and ethnicity) both in UK academic institutions and in the NHS (1, 23). Whilst there has been an improvement in the representation of CA, more work still needs to be done to attract and importantly sustain a diverse population of CA.

It is critical to understand the negative and positive experience of doctors and dentists from minoritised backgrounds, the barriers they experience and areas of support and institutional good practice in removing any systemic barriers. This data will inform recommendation at a local level which will also be applicable to a wider UK context.

For peer review only - http://bmjopen.bmj.com/site/about/guidelines.xhtml

METHODS

Design

A qualitative study using semi-structured interviews and focus groups was conducted to explore the experiences of the CA doctors and dentists in Greater Manchester, with a particular focus on those identifying as being from minoritised backgrounds (including but not limited to gender, race/ethnicity, and/or sexual orientation).

Setting and samples

Theoretical and snowball sampling were employed to recruit CA (doctors and dentists, in research-focused, teaching-focused, and research and teaching posts) and medical and dental students (years 3-5) from the University of Manchester, clinicians in Greater Manchester, key senior stakeholders and gatekeepers to CA and clinical posts in Greater Manchester. The aim of this study was to understand the experiences of people at different stages of CA career pathway. Therefore, a wide range of participants from students to senior academics/clinicians were included. This generated diversity of thought in developing an understanding of the barriers and enablers that influenced the decision to pursue a CA career pathway. The aim of theoretical sampling is to "generate theory whereby the analyst jointly collects, codes and analyses his data and decides what data to collect next and where to find them in order to develop his theory as it emerges" (24). Snowball sampling is a technique where existing participants provide referrals of potential participants interested in taking part in the study (25). In order to capture the diversity of experiences, participants were recruited from a wide range of health settings, at various stages of their careers, and with an emphasis to include minoritised profiles.

Data collection

Potential participants were identified through the members of established medical education networks, clinical academic training and academic networks of the research team, and the snowball sampling approach involving already interviewed participants. Posters and social media platforms (such as Twitter and Faculty newsletters at the University of Manchester) were used to advertise the study. Any potential participants who were interested in taking part were able to contact the authors directly. All potential participants were provided with an online information sheet and gave fully informed consent before and during participation in the

For peer review only - http://bmjopen.bmj.com/site/about/guidelines.xhtml

research. The research was designed to take into account the busy schedule of clinical academics, clinicians, and stakeholders, and the participants were given the option to choose between individual semi-structured interviews and focus groups. All participants, except the students, choose the individual interview because it was easier to fit in their schedule. Interviews topic guides for semi-structured individual interviews and focus groups are available as supplemental files (Supplement 1).

Previous studies have shown Information power (26) to be important when the intention is to collect a rich account of experiences of participants. This means that the more information the sample holds (such as that provided with the in-depth interviews in the present study) the desired information power is achieved with fewer participants. The concept of information power focuses on how to accurately represent participants views, generate in-depth data, and cover the variability of relevant events (26). This study was guided by the principle of information power, as the research team aimed to include participants from different stages of the CA career pathway.

Both focus group and individual interviews were carried out virtually through Zoom [5.13, Zoom Communications] or Microsoft Teams [1.5, Microsoft], lasting between 17 and 61 minutes. The interview topics included experiences of CA career pathway or stakeholder perspective, barriers/facilitators encountered, especially for people from minoritised groups, and experiences of local research cultures. Brief field notes were taken for all interviews and focus groups to facilitate data analysis and identify the initial coding. All interviews and focus groups were conducted by first two authors (CL & CG). Data collection was undertaken from September 2022 to February 2023.

Data analysis

All interviews and focus groups were digitally recorded, transcribed and analysed following Braun and Clarke's reflexive approach to thematic analysis (27). Further, to consider existing qualitative research on the careers of CA and the barriers faced by minoritised groups, the interpretation of the results was integrated with the principle of 'reconstruction' from the extended case method proposed by Burawoy, in which anomalies in relation to an established theoretical corpus are considered and integrated in the analysis through the addition of new themes (28).

For peer review only - http://bmjopen.bmj.com/site/about/guidelines.xhtml

RESULTS

The study aimed to explore the experiences, barriers, and facilitators that CA (doctors and dentists) encounter in pursuing a CA career. A total of 43 participants took part in either an interview (37 clinical academic, clinician, and stakeholder participants) or a focus group for this study (6 medical or dental student participants). In this study, 21 men and 22 women with a wide range of roles participated (19 clinical academics, 5 clinicians, 13 stakeholders and 6 medical or dental students). The 6 students were all in their 20s, the other participants were aged between 30 and 70 (9 in 30-39; 7 in 40-49; 18 in 50-59 and 3 in 60+). Of the 43 participants, 32 were White, 9 were Asian, and 2 were Black. The semi-structured interview framework focused on both the barriers and facilitators for minoritised groups and also general barriers and facilitators applicable to all CA.

Six main themes were generated: Theme 1: A highly competitive pathway that gives limited job security; Theme 2: Balancing two high-workload careers; Theme 3: Lack of representation and role models as limits to belonging; Theme 4: Being foreign or perceived as such; Theme 5: Childcare and gender inequalities; Theme 6: Support and recognition through mentorship. Of these themes, the first three describe barriers common to all CA, themes 3-5 describe barriers that impact in particular participants from minoritised groups, while theme 6 describes one of the main facilitating factors for all CA. Details of themes and sub-themes are summarised in table 1.

Table 1: themes and sub-themes

Theme	Sub-theme
A highly competitive pathway that gives limited job security	Financial challenges
	Jobs insecurity
	Limited funding
Balancing two high-workload careers	Heavy workload
	Limited time for conducting research
Lack of representation and role models as limits to belonging	Diverse role models
	Support from peers with a similar background

For peer review only - http://bmjopen.bmj.com/site/about/guidelines.xhtml

Being foreign or perceived as such	A feeling of belonging
	A language barrier
Childcare and gender inequalities	Maternity leave
	Parenting responsibility
Support and recognition through mentorship	Supportive mentorship from peers with similar experience
	Useful advice from mentors

Theme 1: A highly competitive pathway that gives limited job security

Participants expressed a strong feeling of insecurity in relation to their own career as CA, describing CA as a more precarious pathway when compared to a clinical career. Participants at all levels of seniority recognised that there is fear while holding fixed-term positions of losing one's jobs, and of being unable to control how long CA employment would be based on the success rate of obtaining grants and the duration of the grants.

Several participants mentioned the *financial challenges* of the CA pathway, including precarity and uncertainty linked to fixed-term contracts and *limited funding*. It was seen as problematic that CA needed to secure funding to continue in their career paths, particularly in the early stages of their career. One gatekeeper said:

> *"There is a lot of career uncertainty in academic life. I think it's becoming increasingly so since, say, in the last decade or so, because of funding difficulties in higher education. Determined junior members of staff may start out enthusiastically trying to cut a path in academic life, but I think they become frustrated at the lack of security in their job and it's all too easy to feel that you could move into a less rigorous discipline."* [G04]

Feelings that they were at risk of *losing their jobs* after the completion of the Academic Clinical Lecturer (ACL) training were widely reported by the participants. One said:

> *"I suppose the key step is if you're at the ACL level either with a formal ACL or trying to get your first independent fellowship, if that doesn't get funded by the time your ACL ends then just to earn a living you have to go back to the NHS, and then I think it's quite*

For peer review only

For peer review only - http://bmjopen.bmj.com/site/about/guidelines.xhtml

difficult. You've got to be very driven to be able to drive forward a fellowship application whilst you're still working full time in the clinical arena." [G07]

One of the privileged routes to enter the CA pathway is through fellowships, and *funding opportunities* for fellowships are extremely competitive. One participant mentioned:

"There's a sort of a wish that everybody will go and get a very prestigious MRC [Medical Research Council] or Wellcome Trust fellowship, but actually there's not enough of those to go around." [G06]

The participants who obtained the most prestigious fellowships and had straightforward careers (e.g. transiting from specialisation to academic clinical fellow and then lecturer progressing on to a permanent CA position) also reported being concerned or anxious about finding their next job or securing their next grant in academia. The participants felt that continuous and secure employment was increasingly difficult to find. Therefore, uncertainty of subsequent employment and funding was also described as a major barrier to staying in the field. For example:

"As opposed to research, where you literally, if you don't have the next thing [research job]*, it's like where is your next pay cheque coming from. So, also making that not a thing for people who are specifically research. I don't know how you would do that. It's just, yeah. I know there's no easy answer to some of these things."* [CA07]

Whilst all participants were keen to pursue CA career pathways, the *uncertainty of employment* as a CA emerged as a constant source of anxiety. As a result, the participants felt that they needed to prioritise and maintain an income to meet their basic living costs. A number of participants believed that a purely clinical career was more attractive due to its stability and clearly defined clinical progression route. One participant also explained:

"Academia does not support a family life, whereas the NHS does so. And when I speak to my peers who are in academia, that's what they say: they are just not supported. And

For peer review only - http://bmjopen.bmj.com/site/about/guidelines.xhtml

the way that academia works, is that you're going from contract to contract and grants, no stability at all. So, I'm glad I'm not an academic for that reason." [G08]

The importance of securing and *sustaining funding* was noted as a key challenge by a number of the participants. Supporting early and mid-career CA fellows through internal and external bridging funds whilst they are preparing for their next grant application was identified as a facilitator for career progression. One mid-career clinical academic shared the following experience:

"*These things* [funding] *are very competitive. I was very fortunate: I got the *** Clinical Training Fellowship, the first I applied for, and I was successful, so that was fortunate. The Intermediate Fellowship took me a few goes and at the time there was no... there were no schemes to support clinical academics in that intermediate phase. And I think it's a bit better now because there's the NIHR clinical lectureship scheme that provides some reaching* [bridging] *support, because you can't jump straight from a clinical train to a PhD and straight to an intermediate fellowship. So, I was very fortunate to be working in an environment where there was funding to support me, um, but that was a challenge."* [CA18]

Theme 2: Balancing two high-workload careers

Clinical academic careers can offer the benefits and rewards of making a difference to patients and having the space to pursue research projects. However, the challenge of how to *balance an academic role with a clinical career* was one of the common barriers mentioned:

"And I say to all clinicians who express an interest in academic work that everything is possible, but that you are embarking upon a career that has a clinical component and academic component, and you will be juggling, and it will affect the intensity of your work. So, people have the awareness that this isn't gonna be the easiest route." [G11]

The workload for both the clinical and academic roles often goes beyond what is anticipated based on the clinical academic job plan. Effective line management and responsive workload

For peer review only - http://bmjopen.bmj.com/site/about/guidelines.xhtml

management between both roles can reduce stress and improve satisfaction whilst meeting the demands of both roles. From participants' reports, *workloads* go beyond the official hours both in the clinic and in academia. In addition, balancing research, teaching, and clinical roles was a common challenge:

> *"Two-and-a-half days were taken up by my clinical work, which is the same as was, and two days were taken up for teaching, so it's not quite accurate to say I had no research time, but I had half a day of research time in that five days and when you are a clinical academic you need to do lots of things to build up your name, especially when you're starting. You need time to go for grants. You need time to write papers. You need time to network and do increasing amounts and gain momentum."* [CA09]

A common difficulty was the feeling of being considered as not doing enough work in the clinic compared to full-time colleagues, with the research work in some cases being considered less strenuous than the clinical work that CA are expected to do in the same measure as full-time colleagues. For junior clinicians this further could mean being potentially seen as someone not acquiring the necessary clinical skills, as in the case of this junior clinical academic:

> *"If we are not in theatre learning, we won't acquire our skills. Particularly from a clinical perspective it requires a lot of flexibility from trainers. These are surgeons, they just live, breathe, eat surgery. I am there 50% of the time. Sometimes they see me, sometimes they don't, and then they ask me 'are you here tomorrow?' You say 'no, I am not here'. You feel inadequate, you feel like you are not giving 100%."* [CA06]

Theme 3: Lack of representation and role models as limits to belonging

Barriers linked to systemic forms of discrimination can act on a more personal level, undermining a person's sense of self-worth and well-being. Sexism, racism, and other forms of discrimination that individuals experience throughout their life can impact their work life.

> *"But as an ethnic minority you feel those, you have grown up with them and some of them are not overt. Prejudices are, for me, I can usually sense them rather than*

For peer review only

> *someone* [...] *overtly saying no* [...] *You have heard it so many times, you kind of just think, right, okay, I am going to go and knock on a different door. I would say those things are the things that made me constantly more think I have to prove myself, I have to prove myself."* [CA06]

The importance of representation and a feeling of belonging is crucial for a diverse and dynamic workforce. However, it is common for some specialties, research fields, and/or senior leadership positions to be occupied by white British men. Many of the women participants have for example described leadership positions within academia as closed circles. Indeed, many of the women who did not think that their gender has negatively impacted on their career recognise that some higher roles are much more male-dominated.

> *"We still live in a world where they* [men] *dominate senior academic and senior clinical and senior college and senior government type positions, and so they make a lot of decisions. I think that they occupy that space more than women, and that is levelling up all the time. I don't think they've ever stood in my way."* [CA11]

The lack of *diverse role models* and peers with whom it was possible to identify was discussed, along with the feeling of being out of place in different settings.

> *"If I go to a meeting of researchers* […] *I might be the only South Asian in the room. That's when I feel like a minority; I never feel like a minority in clinical meetings."* [CA09]

> *"And even less so when you're black and woman, there's even less of that* [ethnic minority women in CA] *And sometimes it's like, oh…can you do this?"* [CA07]

Greater Manchester is arguably one of the most diverse areas of the UK. The importance of diversifying the CA pipeline was recognised in the Trust's Equality, Diversity, and Inclusion Strategy 2019-2023 (introduced by Manchester University NHS Foundation Trust) (22) which led to actions such as improving diversity of interview panels. Some interviewees reported positive experiences, such as this female ethnic minority CA:

For peer review only - http://bmjopen.bmj.com/site/about/guidelines.xhtml

So, I went and did the interview and my interview panel actually was a group of all women, all different ethnicities and, I don't know, immediately I just felt way more at ease with them. I felt like I could be myself. We spent 45 minutes talking about me and my career goals and what I wanted and they really…I felt like they really got to know me and they gave me that time and respect to open up and answer questions. [CA04]

Theme 4: Being foreign or perceived as such

Along with the difficulties linked to race and ethnicity, some of the barriers discussed by the interviewees were more specifically linked to not being British-born or not being British citizens. Having a foreign accent was recognised as a marker of not being British-born, and potentially non-British-trained. As a result, CA with the same ethnicity could have very different experiences. As one participant discusses:

"The other thing I think is something to do with how well you can speak and whether you have an unusual accent, which some people do, sometimes that accent can be taken as a bias against subconsciously, I feel. It hasn't happened to me, but I have seen other people who are very capable, but with a strong accent […] *maybe could have done better."* [C04]

Language barriers, even if very modest, were also recognised as potentially creating a disadvantage in publishing and therefore building esteem and an academic career. Others underlined the role that discrimination can play, such as the peer review of grants and articles. Interviewees have underlined how in these cases the doubt remains whether a non-English name might negatively influence the outcome.

Theme 5: Childcare and gender inequalities

The most discussed barriers experienced along gender lines were linked to maternity and childcare and return to work following a period of maternity leave was described as difficult by a number of interviewees, particularly where the expectation was that work can resume without any disruptions.

For peer review only - http://bmjopen.bmj.com/site/about/guidelines.xhtml

> *"The one thing* [a supervisor] *said to me was 'you just need to make sure that your childcare is bomb-proof'. So, I don't know how you can manage to have childcare that is completely perfect."* [G11]

t

Although maternity leave is recognised when accounting for career breaks in the academic CV, a number of interviewees felt that interruptions for maternity were not taken into account when considering the track record of candidates for promotion and career progression. *Maternity* was recognised as a source of discrimination even by women who did not have children. One stakeholder observed:

> *"I think [that] for women [it is difficult] to progress because of the time barrier. If you're then having children and you're trying to juggle a clinical academic career, it's almost like you have two full time jobs… So, we lose a lot of women, and that's why you tend to see a lot of male clinicians who are then on the research path."* [G12]

Childcare was further linked by several participants to barriers for the CA careers. A number of interviewees discussed how childcare continues to largely fall upon women, and how it is difficult to reconcile the already high workload of clinic, research and/or teaching with parenting. The difficulty of managing the different workloads combined with the demands in terms of flexibility and potential need to relocate linked to the academic career. As a result, a number of interviewees observed how a purely clinical career was more attractive as it was more stable with less workload outside hospital hours, and how this might be an incentive to abandon the CA pathway.

Theme 6: Support and recognition through mentorship

Mentorship was identified as a main facilitator for CA career development. Mentorship relationships were mostly built through personal contacts, professional networks, or formal mentorship programs. Typically, the participants were more likely to seek mentorship from people who have the same or similar life experiences and were successful in CA. The participants believed that supportive mentorship could facilitate exploring their research

For peer review only

For peer review only - http://bmjopen.bmj.com/site/about/guidelines.xhtml

interests, building their portfolio and expanding their professional networks. Mentors were able to provide deep insight into CA career pathways, for example:

> *"The person who's helped me the most was my PhD supervisor. He is a clinical academic; he interviewed me the first time round and didn't give me the fellowship, but encouraged me to seek other opportunities. He knew… because he'd been through it himself, he was aware of the trajectory, he was aware of what was needed, he really helped me in terms of how to write a fellowship application, how to sell yourself, really, which I think is something that I struggle with. So he's been very instrumental in helping my career, definitely, and seeing that I potentially have a future in this."* [CA03]

Participants who self-identified as earlier career researchers, pointed out the importance of *senior and peer mentors* in CA career pathways. Beyond the academic support, some participants also noted that mentors are pivotal when they encounter challenges in the field. The benefits of mental or emotional support from these mentors were reported. Based on their advanced knowledge or experience, mentors were in a good position to offer *advice on CA career pathways*. The participants felt they were protected: in a safe place. They believed these mentors could help them to develop into professional and independent CA, for example:

> *"I had a fantastic mentor* […] *and she kind of took me under her wing. She could see I felt a bit lost. I didn't get the training number. I was, you know, five hours away from my family and she kind of just gave me this motivation. And some focus to say, 'Right. You're next. I know it's difficult for you up here and you're lonely, but you need to focus on the next step.' And she was pivotal really, in that she really believed in me. Like, really, for somebody, I didn't know very well. I don't know. She had this incredible ability to make me quite confident in my skills."* [CA06]

DISCUSSION

The participants to our research project form a diverse group including CA, clinicians, students and stakeholders, which has made it possible to understand a wide range of difficulties and problems impacting decision-making and career choices. Six themes have been identified, with

For peer review only - http://bmjopen.bmj.com/site/about/guidelines.xhtml

two describing general barriers experienced by CA, three capturing further the complex experiences of minoritised groups, and one identifying an important facilitator.

This project has corroborated previous research (9, 10) in showing the difficulties in managing two demanding careers and in finding a balance for activities with a significant workload that are difficult to scale to part-time positions, and how this creates challenges for career advancement, especially for people with childcare responsibilities.

Both this study and previous research reported that various challenges such as job insecurity and heavy workload could be key barriers to pursuing a clinical academic career (9-13). Several new elements emerge from the analysis of our data. Among the general barriers, our participants mentioned recent changes in the career pathway, including the reduction of available positions, coupled with the expectation of obtaining prestigious fellowships before advancing in CA careers.

The participants highlighted the degree to which obtaining such fellowships requires time, support from experienced colleagues, and availability of bridge funding to allow time to re-apply in case of rejection. While available in the context studied, such resources are unequally distributed, and require in turn a significant time and financial investment for an uncertain outcome. In particular, support from peers and colleagues, particularly at a senior level, were recognised as a key facilitator to tackle these challenges, both in our study and in previous literature (10, 11). A recent systematic review of the existing interventions to support clinical academic careers for doctors and dentists found that protected research time could enhance CA retention and career progression (11). In addition, to bolster the feeling of belonging for minoritised CA, representation and inclusion is crucial.

One of the main individual barriers are the ways in which being minoritised hinders one's confidence. Ethnic minority participants reported episodes of racism, in their careers, but further discussed how experiences of discrimination and racism suffered in other spheres of life can have an impact also on their self-worth in a professional setting. A strong sense of being an "outlier" is still commonly reported from women and minority populations in the research environment in the literature (9,13-15). This might explain the feeling of unbelonging

For peer review only - http://bmjopen.bmj.com/site/about/guidelines.xhtml

that further erodes confidence. For example, women participants perceived men occupying the majority of the senior and management positions as disempowering. Despite improvement in female representation in leadership positions, gender imbalance can impact career progression through the phenomena already identified as glass ceiling and/or sticky floors (9, 10). Ethnic minority participants have reported similar experiences, with further issues such as having a name perceived as foreign and being potentially disadvantaged in being evaluated for publications and grants.

Taken together these data highlight the range of systemic barriers experienced by minoritised CA. These ranged from how having an accent was perceived as limiting progression, to lack of role models, difficulties in balancing childcare commitments and two highly demanding roles. The study also highlighted key enablers through bridging financial support, grant writing support and recognising lived experience and supporting career progression through mentorship. These findings will help inform individuals, senior leaders and organisations of the facilitators and barriers that impact on CA career progression and help policy development for those leading on clinical academic training programmes and delivery of a targeted equality and diversity strategy.

There are limitations to this study: the fact that only a quarter of the respondents were from minoritised groups should be taken into account in the analysis of the results. Further studies with wider and more diverse samples might be helpful to enhance transferability in qualitative research. Meanwhile, as one aim of this study was to understand the experiences of minoritised groups, the composition of the research team should also be considered. Our team is diverse in terms of ethnicity and national origin, but is entirely female, which can have had a role in the collection and analysis of the data.

CONCLUSIONS

The study showed that there are several barriers that current and prospective CA face. It has also shown that barriers and difficulties are increased for participants from minoritised groups. To remove these barriers, cultural and structural interventions are needed. On the one hand, it is important to identify forms of financial support for early career CA, in order to make the CA pathway more financially stable. On the other hand, it is important to encourage wider social

For peer review only - http://bmjopen.bmj.com/site/about/guidelines.xhtml

and cultural changes that can reduce the feelings of unbelonging that women and ethnic minority participants in particular have expressed. These findings and recommendations are of relevance for senior leaders and organisations involved in clinical academic training and more general in equality & diversity and careers strategy.

For peer review only

Acknowledgments

Thanks to Prof Colette Fagan and Prof Adam Danquah, University of Manchester for helpful discussions during the course of this project. Thanks to clinicians, clinical academics, students and stakeholders who participated or facilitated this study.

Contributors

HR and NG conceived of and led the study as principal investigators; CL and CG conducted data collection and data analysis; GF, HR and NG contributed qualitative data analysis. All authors (CL, CG, HR, GF, RC and NG) made contributions to the manuscript and reviewed the final submission.

Funding: This work was supported by University of Manchester and Wellcome matched funding reference 204796/Z/16/Z ISSF - Wellcome ISSF 3 - EDI.

Declaration of Interest: None

Patient and public involvement

Patients and/or the public were not involved in the design, or conduct, or reporting, or dissemination plans of this research.

Patient consent for publication

Not applicable.

Ethics approval

This study involves human participants and was approved by the University of Manchester Ethics Committee (Reference: 2022-13791-23819). Written informed consent was obtained from all participants in the study before taking part.

Data availability statement

The interview and focus group data used in this study are not publicly available due to integrity statements in the ethics approvals.

For peer review only - http://bmjopen.bmj.com/site/about/guidelines.xhtml

REFERENCES

1. Medical Schools Council. Clinical academic survey [Available from: https://www.medschools.ac.uk/clinical-academic-survey accessed 10/04 2023.
2. Nicole W. Survey of Medical Clinical Academic Staffing Levels London: Medical Schools Council; 2018 [Available from: https://www.medschools.ac.uk/media/2491/msc-clinical-academic-survey-report-2018.pdf2023.
3. Linden JA, Baird J, Madsen TE, et al. Diversity of leadership in academic emergency medicine: Are we making progress? *The American Journal of Emergency Medicine* 2022;57:6-13. doi: https://doi.org/10.1016/j.ajem.2022.04.009
4. Richter KP, Clark L, Wick JA, et al. Women physicians and promotion in academic medicine. *New England Journal of Medicine* 2020;383(22):2148-57. doi: 10.1056/NEJMsa1916935
5. Parker RB, Stack SJ, Schneider SM. Why diversity and inclusion are critical to the American college of emergency physicians' future success. *Annals of Emergency Medicine* 2017;69(6):714-17. doi: 10.1016/j.annemergmed.2016.11.030 [published Online First: 2017/02/01]
6. Alsan M, Garrick O, Graziani G. Does diversity matter for health? Experimental evidence from Oakland. *American Economic Review* 2019;109(12):4071-111. doi: 10.1257/aer.20181446
7. Beebeejaun K, Littleford K. A diverse public health workforce is more important than ever. *BMJ (Clinical research ed)* 2023;380:p447. doi: 10.1136/bmj.p447
8. Marmot M. Building back fairer in Greater Manchester and the country. Royal Society Open Science 2021;8(10):211454. doi: doi:10.1098/rsos.211454
9. Ranieri VF, Barratt H, Rees G, et al. A qualitative study of the influences on clinical academic physicians' postdoctoral career decision making. *Academic Medicine* 2018;93(11):1686-93. doi: 10.1097/acm.0000000000002141 [published Online First: 2018/01/26]
10. Trusson D, Rowley E, Bramley L. A mixed-methods study of challenges and benefits of clinical academic careers for nurses, midwives and allied health professionals. *BMJ Open* 2019;9(10):e030595. doi: 10.1136/bmjopen-2019-030595
11. Raine G, Evans C, Uphoff EP, et al. Strengthening the clinical academic pathway: a systematic review of interventions to support clinical academic careers for doctors and dentists. *BMJ Open* 2022;12(9):e060281. doi: 10.1136/bmjopen-2021-060281
12. Kehoe A, Crampton P, Buchanan J, et al. Tips to Support the Recruitment, Retention, and Progression of Clinical Academics. *Medical Science Educator* 2022;32(2):503-09. doi: 10.1007/s40670-022-01512-1
13. Finn G, Morgan J. From the sticky floor to the glass ceiling and everything in between: A systematic review and qualitative study focusing on gender inequalities in Clinical Academic careers, 2020.
14. Vassie C, Smith S, Leedham-Green K. Factors impacting on retention, success and equitable participation in clinical academic careers: a scoping review and meta-thematic synthesis. *BMJ Open* 2020;10(3):e033480. doi: 10.1136/bmjopen-2019-033480
15. Woolf K, Rich A, Viney R, et al. Perceived causes of differential attainment in UK postgraduate medical training: a national qualitative study. *BMJ Open* 2016;6(11):e013429. doi: 10.1136/bmjopen-2016-013429
16. Finn GM, Crampton P, Buchanan JA, et al. The impact of the COVID-19 pandemic on the research activity and working experience of clinical academics, with a focus on gender

For peer review only - http://bmjopen.bmj.com/site/about/guidelines.xhtml

and ethnicity: a qualitative study in the UK. *BMJ Open* 2022;12(6):e057655. doi: 10.1136/bmjopen-2021-057655
17. Finn GM, Crampton PES, Kehoe A, et al. Will removing intercalation undermine our clinical academic foundations? *BMJ (Clinical research ed)* 2021;374:n1527. doi: 10.1136/bmj.n1527
18. Boatright DH, Samuels EA, Cramer L, et al. Association between the Liaison Committee on Medical Education's Ddversity standards and changes in percentage of medical student sex, race, and ethnicity. *Jama* 2018;320(21):2267-69. doi: 10.1001/jama.2018.13705 [published Online First: 2018/12/05]
19. Priest N, Esmail A, Kline R, et al. Promoting equality for ethnic minority NHS staff—what works? *BMJ : British Medical Journal* 2015;351:h3297. doi: 10.1136/bmj.h3297
20. Ovseiko PV, Taylor M, Gilligan RE, et al. Effect of Athena SWAN funding incentives on women's research leadership. *BMJ (Clinical research ed)* 2020;371:m3975. doi: 10.1136/bmj.m3975
21. Kline R, Naqvi H, Razaq SA, et al. NHS Workforce Race Equality Standard: 2016 Data Analysis Report for NHS Trusts 2017 [Available from: https://www.england.nhs.uk/wp-content/uploads/2017/03/workforce-race-equality-standard-data-report-2016.pdf accessed 20 May 2023.
22. Equality, Diversity and Inclusion: Manchester Foundation Trust; 2023 [Available from: https://mft.nhs.uk/the-trust/equality-diversity-and-inclusion/ accessed 10/04 2023.
23. NHS Workforce Race Equality Standard 2022 [Available from: https://www.england.nhs.uk/wp-content/uploads/2022/04/Workforce-Race-Equality-Standard-report-2021-.pdf accessed 10/04 2023.
24. Glaser BG, Strauss AL. *The Discovery of Grounded Theory: Strategies for Qualitative Research*. 2017 Routledge.
25. Parker C, Scott S, Geddes A. . Snowball Sampling, In P Atkinson, S Delamontet al. (Eds.), *SAGE Research Methods Foundations*. 2019. https://doi.org/10.4135/9781526421036831710
26. Malterud K, Siersma VD, Guassora AD. Sample Size in Qualitative Interview Studies: Guided by Information Power. *Qualitative Health Research* 2016;26(13):1753-1760. doi: 10.1177/1049732315617444
27. Braun V, Clarke V. Reflecting on reflexive thematic analysis. *Qualitative Research in Sport, Exercise and Health* 2019;11(4):589-97. doi: 10.1080/2159676X.2019.1628806
28. Burawoy, M. The Extended Case Method. *Sociological Theory* 1998;16(1): 4-33. doi: 10.1111/0735-2751.00040

For peer review only

Supplement 1:

Indicative interview question stems – SCALE

Please note, the following are suggested, iterative question stems. The group interviews and individual interviews will both be semi-structured in nature, so interviewers will also be able to follow interesting lines of questioning/prompt further than the lists supplied below, if necessary. Interviewers will meet regularly to discuss interesting insights from the interviews, and will have the agency and ability to modify, evolve or add questions as appropriate, and as befits the iterative design of this qualitative research

Clinician academics focus groups

1. Tell us about your journey into clinical academia (Icebreaker question).
2. What facilitated your journey?
    a. (If appropriate): Why do you think you experienced such facilitators?
3. Did you experience any difficulties or barriers to your career?
    a. If so, please elaborate.
    b. Why do you think you experienced these barriers?
4. What is the research culture of your academic institution like?
5. Has the culture you've experienced at your academic institution influenced any decisions you've made about your career?
    a. If so, how?
6. Has the culture you've experienced within medicine/dentistry and academia more generally influenced any decisions you've made about your career?
    a. If so, how?
7. Thinking about any barriers or negative experiences of culture that have been mentioned, what changes could be made on an institutional level to enable clinical academics from minoritised backgrounds to flourish?
8. Is there anything else you wish to tell us about your experiences of clinical academia within Greater Manchester?

Clinicians focus groups

1. Tell us about your journey into clinical practice to date (Icebreaker question).
2. What facilitated your journey?
    a. (If appropriate): Why do you think you experienced such facilitators?
3. Did you experience any difficulties or barriers to your career?
    a. If so, please elaborate.
    b. Why do you think you experienced these barriers?
4. How do you view careers in clinical academia?
    a. Does anyone have experience as a clinical academic? If you left an academic post, can you tell us why?
    b. Have you ever considered a clinical academic career? If the answer is yes but you chose not to pursue a clinical academic career, can you tell us why?
    c. For those that haven't considered clinical academic careers, can you tell us why? Did anything in particular put you off?

For peer review only

5. In Greater Manchester more specifically, do you get any sense of what it's like to be a clinical academic?
    a. What do you think about the culture surrounding research in Greater Manchester?
    b. Have your experiences or perceptions of this culture influenced the decisions you've made about your career in any ways?
6. Thinking about any barriers or negative experiences of culture that have been mentioned, what changes could be made on an institutional level to enable clinicians from minoritised backgrounds to pursue clinical academic careers, if they wish?
7. Is there anything else you wish to tell us about your experiences of the research culture or academia within Greater Manchester?

Medical and dental students focus groups

1. How familiar are you with the clinical academic career pathway? (Icebreaker question).
2. What kind of information, if any, have you received about clinical academic career during your studies?
3. Would you be interested in pursuing an academic career along with your clinical one?
4. Do you have an opinion about intercalated degrees?
5. Do you see difficulties or barriers to start a clinical academic career?
    a. If so, please elaborate.
    b. What do you think is the reason behind these barriers?
6. What is the institutional culture of the University of Manchester like?
7. Has the culture you've experienced at the university influenced any plans you've made about your career?
    a. If so, how?
8. Thinking about any barriers or negative experiences of culture that have been mentioned, what changes could be made on an institutional level to enable people from minoritised backgrounds to start a clinical academic career?
9. Is there anything else you wish to tell us about your experiences within the University of Manchester?

Gatekeeper interviews

1. Can you tell me about your current role? (Icebreaker question)
    a. Do you have/ What responsibilities do you have in terms of managing clinicians/clinical academics?
    b. Do you have/ What responsibilities do you have in terms of making selection decisions in clinical academia?
2. What have your experiences of managing clinicians/clinical academics and/or making selection decisions been like?
3. From your perspective, what are the facilitating factors for clinicians looking to build clinical academic careers?
4. From your perspective, what are the barriers for clinicians looking to build clinical academic careers?

For peer review only

5. Do you feel able to support the staff you manage to confront or tackle the barriers they may experience?
    a. If yes, can you give some examples of what this support looks like in practice? What resources does this support require?
    b. If no, can you please explain why?
6. If you have experience making selection decisions, do you feel able to confront any barriers to clinical academic careers in your role within selection?
    a. If yes, how?
    b. If no, why not? Could anything change to enable you to challenge the barriers you are aware of?
7. Thinking specifically about the experiences of those you manage who are from minoritised backgrounds, are there any additional facilitating factors for minoritised clinicians looking to build clinical academic careers?
8. Thinking specifically about the experiences of those you manage who are from minoritised backgrounds, are there any additional barriers for minoritised clinicians looking to build clinical academic careers?
9. Do you feel able to support the staff you manage from minoritised backgrounds to confront or tackle the barriers they may experience?
    a. If yes, can you give some examples of what this support looks like in practice? What resources does this support require?
    b. If no, can you please explain why?

For peer review only

Standards for Reporting Qualitative Research (SRQR)*

http://www.equator-network.org/reporting-guidelines/srqr/

Title and abstract

	Page/line no(s).
**Title** - Concise description of the nature and topic of the study Identifying the study as qualitative or indicating the approach (e.g., ethnography, grounded theory) or data collection methods (e.g., interview, focus group) is recommended	Page 1
**Abstract** - Summary of key elements of the study using the abstract format of the intended publication; typically includes background, purpose, methods, results, and conclusions	Page 1

Introduction

**Problem formulation** - Description and significance of the problem/phenomenon studied; review of relevant theory and empirical work; problem statement	Page 3
**Purpose or research question** - Purpose of the study and specific objectives or questions	Page 4

Methods

**Qualitative approach and research paradigm** - Qualitative approach (e.g., ethnography, grounded theory, case study, phenomenology, narrative research) and guiding theory if appropriate; identifying the research paradigm (e.g., postpositivist, constructivist/ interpretivist) is also recommended; rationale**	Page 4-5
**Researcher characteristics and reflexivity** - Researchers' characteristics that may influence the research, including personal attributes, qualifications/experience, relationship with participants, assumptions, and/or presuppositions; potential or actual interaction between researchers' characteristics and the research questions, approach, methods, results, and/or transferability	Page 15
**Context** - Setting/site and salient contextual factors; rationale**	Page 4-5
**Sampling strategy** - How and why research participants, documents, or events were selected; criteria for deciding when no further sampling was necessary (e.g., sampling saturation); rationale**	Page 4-5
**Ethical issues pertaining to human subjects** - Documentation of approval by an appropriate ethics review board and participant consent, or explanation for lack thereof; other confidentiality and data security issues	Page 16
**Data collection methods** - Types of data collected; details of data collection procedures including (as appropriate) start and stop dates of data collection and analysis, iterative process, triangulation of sources/methods, and modification of procedures in response to evolving study findings; rationale**	Page5

For peer review only - http://bmjopen.bmj.com/site/about/guidelines.xhtml

**Data collection instruments and technologies** - Description of instruments (e.g., interview guides, questionnaires) and devices (e.g., audio recorders) used for data collection; if/how the instrument(s) changed over the course of the study	Page 5
**Units of study** - Number and relevant characteristics of participants, documents, or events included in the study; level of participation (could be reported in results)	Page 6
**Data processing** - Methods for processing data prior to and during analysis, including transcription, data entry, data management and security, verification of data integrity, data coding, and anonymization/de-identification of excerpts	Page 5
**Data analysis** - Process by which inferences, themes, etc., were identified and developed, including the researchers involved in data analysis; usually references a specific paradigm or approach; rationale**	Page 6
**Techniques to enhance trustworthiness** - Techniques to enhance trustworthiness and credibility of data analysis (e.g., member checking, audit trail, triangulation); rationale**	Page 5

Results/findings

**Synthesis and interpretation** - Main findings (e.g., interpretations, inferences, and themes); might include development of a theory or model, or integration with prior research or theory	Page 6
**Links to empirical data** - Evidence (e.g., quotes, field notes, text excerpts, photographs) to substantiate analytic findings	Page 6-14

Discussion

**Integration with prior work, implications, transferability, and contribution(s) to the field -** Short summary of main findings; explanation of how findings and conclusions connect to, support, elaborate on, or challenge conclusions of earlier scholarship; discussion of scope of application/generalizability; identification of unique contribution(s) to scholarship in a discipline or field	Pag 14-15
**Limitations** - Trustworthiness and limitations of findings	Page 15

Other

**Conflicts of interest** - Potential sources of influence or perceived influence on study conduct and conclusions; how these were managed	Page 17
**Funding** - Sources of funding and other support; role of funders in data collection, interpretation, and reporting	Page 17

*The authors created the SRQR by searching the literature to identify guidelines, reporting standards, and critical appraisal criteria for qualitative research; reviewing the reference lists of retrieved sources; and contacting experts to gain feedback. The SRQR aims to improve the transparency of all aspects of qualitative research by providing clear standards for reporting qualitative research.

For peer review only

For peer review only - http://bmjopen.bmj.com/site/about/guidelines.xhtml

**The rationale should briefly discuss the justification for choosing that theory, approach, method, or technique rather than other options available, the assumptions and limitations implicit in those choices, and how those choices influence study conclusions and transferability. As appropriate, the rationale for several items might be discussed together.

Reference:

O'Brien BC, Harris IB, Beckman TJ, Reed DA, Cook DA. **Standards for reporting qualitative research: a synthesis of recommendations.** *Academic Medicine*, Vol. 89, No. 9 / Sept 2014 DOI: 10.1097/ACM.0000000000000388

For peer review only

For peer review only - http://bmjopen.bmj.com/site/about/guidelines.xhtml
